# A blood gas parameter–based assessment model for predicting poor prognosis in sepsis: A retrospective analysis of the MIMIC-IV and eICU-CRD

**DOI:** 10.1371/journal.pone.0346532

**Published:** 2026-07-09

**Authors:** Xiao Chen, Huichang Zhuo, Yunpiao Wang, Daxuan Wang, Xiaoqin Li, Jiandong Lin, Xiuyu Liao, Xian Lin, Xiao Lin

**Affiliations:** 1 Department of Intensive Care Unit, The First Affliated Hospital of Fujian Medical University, Fuzhou, Fujian, China; 2 Department of Intensive Care Unit, National Regional Medical Center, Binhai Campus of the First Affliated Hospital, Fujian Medical University, Fuzhou, Fujian, China; 3 School of Nursing and Medicine, Minjiang Teachers College, Fuzhou, Fujian, China; 4 Shengli Clinical Medical College of Fujian Medical University, Fujian Provincial Hospital, Fuzhou University Affiliated Provincial Hospital, Fuzhou, Fujian, China; 5 Shenzhen Key Laboratory of Immunity and Inflammatory Diseases, Peking University Shenzhen Hospital, Shenzhen Peking University-The Hong Kong University of Science and Technology Medical Center, Shenzhen, Guangdong, China; Pescara General Hospital, ITALY

## Abstract

**Background:**

Blood gas parameters are associated with sepsis prognosis. This study aimed to develop an assessment model based on blood gas parameters for predicting patient outcomes.

**Methods:**

Data were retrospectively extracted from the Medical Information Mart for Intensive Care IV (MIMIC-IV) and electronic Intensive Care Unit Collaborative Research Database (eICU-CRD). A sepsis assessment model was developed using the MIMIC-IV cohort, followed by internal validation in patients with septic shock from the same database and external validation in patients with sepsis from the eICU-CRD. Bioinformatics and machine learning clarified the relationship between the model and the primary outcome of 28-day mortality in patients with sepsis and septic shock.

**Results:**

The sepsis assessment blood gas 3 (SABG-3), an assessment model incorporating PO_2_, base excess (BE), and lactate, was developed and validated as an independent predictor of 28-day mortality in patients with MIMIC-IV sepsis (odds ratio: 1.559; 95% confidence interval: 1.464–1.659; *P* < 0.001). Its prognostic performance was internally validated in patients with MIMIC-IV sepsis and externally validated in patients with eICU-CRD sepsis. High-risk patients identified by SABG-3 exhibited greater illness severity than low-risk ones. Sensitivity analyses across five methods confirmed the prognostic value of SABG-3 for intensive care unit patients with sepsis and septic shock. A SABG-3-derived nomogram proved superior to existing scales.

**Conclusion:**

We developed and validated a novel assessment model and nomogram to evaluate sepsis prognosis rapidly and to identify patients who may benefit from intensified treatment.

## Introduction

Sepsis, an infection-related disease, manifests via organ dysfunction, dysregulated inflammation, and abnormal immune responses. It causes approximately 20% of deaths worldwide [1–3]. Despite recent declines in mortality, sepsis remains the primary challenge in the intensive care unit (ICU) [4]. Current prognostic assessment scales for sepsis are relatively complicated, relying on scoring systems based on multiple indicators. Thus, identifying a simple and convenient assessment tool that can rapidly evaluate patients would significantly help clinicians decide when to implement intensified treatments, including the Early Management Bundle for Severe Sepsis and Septic Shock [5].

Tools such as the sequential organ failure assessment (SOFA), systemic inflammatory response syndrome (SIRS), simplified acute physiology score II (SAPS II), Oxford acute severity of illness score (OASIS), logistic organ dysfunction system (LODS), and acute physiology score III (APS III) have been widely used to assess sepsis [6,7]. Most of these scores incorporate specific blood gas parameters for risk stratification. Prognostic value in patients with septic shock has been shown in arterial and venous blood gas levels, along with the differences between peripheral arterial and venous blood gas levels [8,9]. Initial, maximum, and mean blood gas values are associated with sepsis prognosis [10,11], and abnormal levels are linked to organ dysfunction [12,13]. However, the prognostic value of a comprehensive set of these parameters has not been systematically investigated, and the effectiveness of using a blood gas–based risk model for prognostication remains unclear.

In the present study, we integrated data from patients with sepsis and septic shock across both databases, Medical Information Mart for Intensive Care IV (MIMIC-IV) and electronic Intensive Care Unit Collaborative Research Database (eICU-CRD), to develop and validate a blood gas parameter–based assessment model. We investigated the association between the proposed sepsis assessment blood gas 3 (SABG-3) model and clinical characteristics to determine its prognostic value in patients with sepsis and septic shock. We further developed a new SABG-3-derived nomogram to identify patients with sepsis who might benefit from intensified therapy.

## Materials and methods

### Data sources

This retrospective study was conducted in accordance with the REporting of studies Conducted using Observational Routinely-collected health data statement [14]. All methods were performed following relevant guidelines and regulations. Patient data were derived from MIMIC-IV version 2.0 and eICU-CRD version 2.0.

### Ethical statement

The authors obtained access to the MIMIC-IV and eICU-CRD databases (Certification No. 37303946). This study was a secondary analysis of de-identified, publicly available data. Thus, ethical approval and informed consent were waived. All procedures adhered to the tenets of the Declaration of Helsinki.

### Data extraction

DataGrip and R software were used for data extraction and processing. Based on a previous report [15], the current study analyzed variables, including age, sex, body mass index (BMI), admission type, race, marital status, service unit, illness severity, interventions, elective surgery, comorbidities, and vital signs within the first 24 h of admission. For variables recorded multiple times within the first 24 h of admission, the value representing the highest level of sepsis severity was selected.

Patient demographics, nursing progress notes, laboratory results, medications, and International Classification of Diseases (ICD) codes were extracted from medical records to identify variables of interest. Sepsis was defined according to the Sepsis 3.0 criteria. Septic shock was identified using ICD codes (785.52 and R65.21). For arterial and venous blood gas tests with multiple measurements [16], the mean, initial, maximum, and minimum values of parameters within the first 24 h of ICU stay were extracted. The arterial or venous difference in blood gas parameters was defined as the maximum value minus the minimum value. Parameters with more than 20% missing data were excluded from analyses, leaving nine blood gas parameters for further examination: alveolar-arterial oxygen gradient (AaDO_2_), base excess (BE), fraction of inspired oxygen (FiO_2_), lactate, oxygenation index (OI), partial pressure of carbon dioxide (PCO_2_), pH, partial pressure of oxygen (PO_2_), and total CO_2_. Patients aged < 18 y were excluded, and only the first ICU admission for each patient with sepsis and available blood gas analyses was included. A total of 13,873 patients with sepsis from the MIMIC-IV database were enrolled as the training cohort. The validation cohort comprised 2,803 ICU patients with septic shock from MIMIC-IV and 3,842 patients with sepsis from eICU-CRD.

### Development of the SABG-3 model using machine learning techniques

The SABG-3 model was developed using a previously described method [17,18] with some modifications. The randomForest package in R was used to rank the prognostic importance of variables. Collinearity analysis was then conducted to identify independent variables among arterial blood gas parameters. Subsequently, univariable logistic regression was performed for descriptive purposes, followed by least absolute shrinkage and selection operator (LASSO) regression with 10-fold cross-validation for variable selection. Finally, multivariable logistic regression was used to optimize the risk model. Patients with sepsis were stratified into high-risk or low-risk groups based on their calculated risk scores. In addition, alternative forms of arterial or venous blood gas parameters were incorporated into separate risk models. Model performance was evaluated using the area under the receiver operating characteristic (ROC) curve (AUC). The optimal cutoff value for the SABG-3 risk score was determined using the maximum Youden index [sensitivity – (1 – specificity)] [19].

### Primary and secondary outcomes

The primary outcome of this study was mortality at 28 d after ICU admission. Secondary outcomes included longer-term mortality (at 90 d and 180 d) and resource utilization metrics within the first 28 d of ICU stay. These metrics consisted of vasopressor and mechanical ventilation requirements, as well as the number of vasopressor-free and mechanical ventilation-free days.

### Statistical analysis

Data were analyzed using SPSS version 21.0 and R software version 4.1.2. For group comparisons, continuous variables were expressed as medians (interquartile ranges) and analyzed using nonparametric tests, or as means (standard deviations) and then analyzed using Student’s t-test. Categorical variables were presented as counts (percentages) and compared using the χ² test.

Kaplan–Meier survival curves were constructed and compared using the log-rank test to evaluate differences between the high-risk and low-risk groups. Time-dependent ROC curves were generated to calculate the AUC. Univariable and multivariable logistic regression analyses were performed to identify independent prognostic factors, with results reported as odds ratios and 95% confidence intervals (CIs).

The robustness of our findings was assessed, and confounders were adjusted via sensitivity analyses based on several previously reported models [20]. These models included a multivariable logistic regression model, a propensity score matching (PSM) model, a propensity score–based inverse probability weighting (IPW) model, a doubly robust model adjusting for unbalanced or all covariates. Subgroup analyses were also conducted. Multivariable analyses were replicated following multiple imputations to minimize potential bias due to missing data. Outliers were treated as missing values.

Collinearity analysis, univariable logistic regression, LASSO regression, multivariable logistic regression, and doubly robust analyses were sequentially applied to construct a prognostic nomogram for predicting individual survival probability in patients with sepsis. The C-index, F1 score, and accuracy (%) were calculated to assess the predictive performance of the nomogram. All statistical tests were two sided, and a *P* value <0.05 was considered statistically significant.

## Results

### Construction of the SABG-3 model in patients with sepsis using the MIMIC-IV database

**Fig 1:**
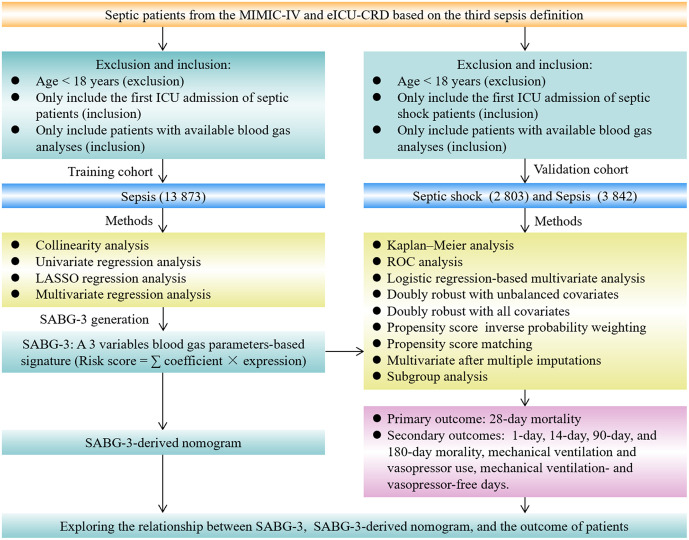
Flow diagram of the study. Overview of inclusion and exclusion criteria, construction of the SABG-3 model, study outcomes, and statistical analyses performed. SABG-3: Sepsis assessment blood gas 3.

The initial search of the MIMIC-IV database yielded 35,010 ICU admissions meeting the Sepsis‑3 criteria. Of these individuals, 13,873 patients with a first ICU admission and available blood gas data were included in the machine learning processes for risk model development (Fig 1).

**Fig 2:**
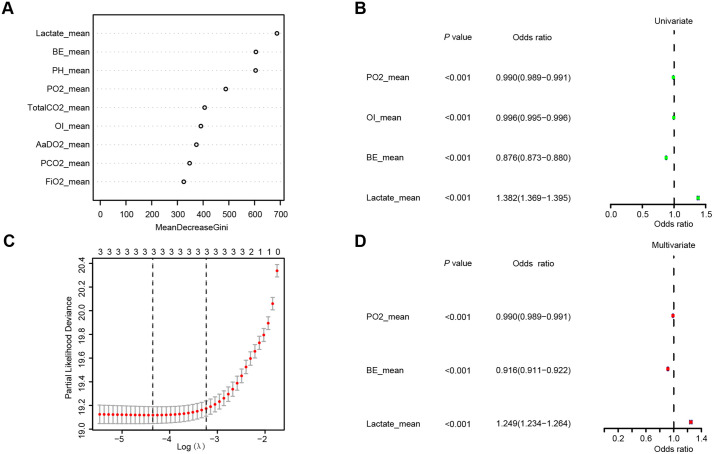
Generation of a blood gas parameter–based assessment model in patients with sepsis. **(A)** Random forest ranking of the importance of nine arterial blood gas parameters. **(B)** Univariable logistic regression illustrating associations of PO_2_, OI, BE, and lactate with 28-day mortality. **(C)** LASSO regression analysis for identifying prominent prognostic variables. (**D**) Multivariable logistic regression confirming the prognostic roles of PO_2_, BE, and lactate in patients with sepsis.

**Fig 3:**
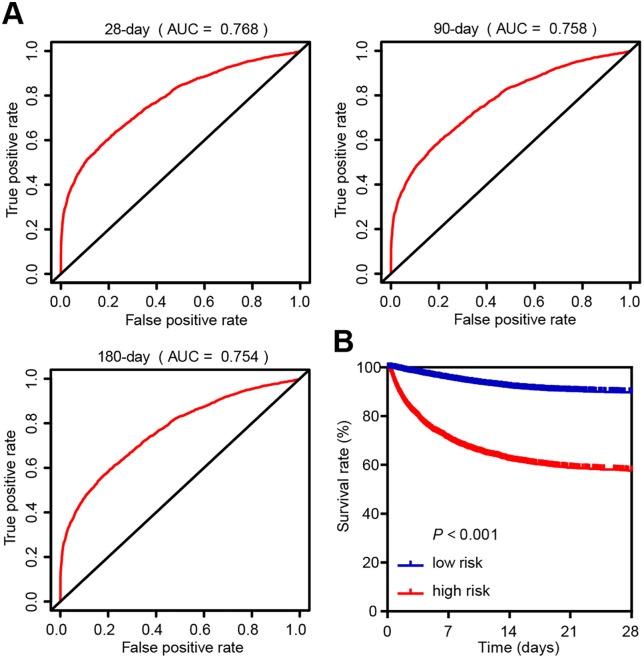
Prognostic performance of SABG-3 in patients with sepsis. **(A)** Time-dependent ROC curves evaluating the predictive performance of the model. **(B)** Kaplan–Meier curves comparing survival between high-risk and low-risk patients stratified by SABG-3, with *P* value determined from the log-rank test. SABG-3: Sepsis assessment blood gas 3.

First, a random forest model was used to rank the importance of nine arterial blood gas parameters (AaDO_2_, BE, FiO_2_, lactate, OI, PCO_2_, pH, PO_2_, total CO_2_) measured within the first 24 h of ICU admission (Fig 2A). Collinearity analysis using a variance inflation factor threshold of < 10 identified OI, PO_2_, BE, and lactate as independent variables. Subsequently, univariable logistic regression analyses demonstrated that OI, PO_2_, and BE had favorable predictive value, whereas lactate was negatively associated with 28-day survival (Fig 2B). LASSO regression was then employed to identify the most prominent prognostic variables, leading to a three-variable prognostic model (Fig 2C). Multivariable logistic regression was used to construct the SABG-3 model comprising PO_2_, BE, and lactate (Fig 2D). The risk score was calculated as follows: risk score = (–0.01021 × PO_2_ values) + (–0.08731 × BE values) + (0.22209 × lactate values) (S1 Table). The risk model based on mean arterial blood gas values from the first 24 h of ICU admission outperformed those constructed using other parameters for predicting 28-day mortality (S2 Table). This finding supports the prognostic value of the mean-value-derived SABG-3 in ICU patients with sepsis (Figs 3A and 3B).

The International Guidelines for the Management of Sepsis and Septic Shock recommend serum lactate measurement for the diagnosis of septic shock [21]. Hence, we evaluated the roles of arterial and venous lactate in assessing sepsis severity. In our established model, arterial lactate was identified as a predictor. In an analysis of blood gas measurements, the AUC of venous lactate was comparable to that of arterial lactate for predicting sepsis prognosis (S1 Fig). These findings suggest that both arterial and venous serum lactate measurements are effective for assessing prognosis in patients with sepsis.

### Demographic data and baseline characteristics

Among the 13,873 patients with sepsis included for the development of SABG-3, 3,669 were assigned to the high-risk group, and the remaining 10,204 to the low-risk group on the basis of the optimal cutoff risk score determined by the ROC curve. Survival was lower in the high-risk group than in the low-risk group (Fig 3B). The 28-day mortality rate was 42.27% (1551/3669) in the high-risk group and 10.25% (1046/10,204) in the low-risk group. Compared with low-risk patients, high-risk ones exhibited greater sepsis severity, characterized by a higher incidence of septic shock, greater illness severity scores, higher requirements for organ support, and more severe vital sign abnormalities. Moreover, the high-risk group included more female, elderly, and unmarried patients. Congestive heart failure, cancer, and liver or renal disease occurred more frequently in the high-risk group. Conversely, the low-risk group had a higher incidence of hypertension, coronary artery disease, and cerebrovascular disease (Table 1).

**Table 1:** Baseline characteristics of septic patients in high-risk and low-risk groups.

Variables	High-risk (n = 3669)	Low-risk (n = 10204)	*P* value
Age (years)	66 (55-77)	65 (55-75)	0.031
Male, n (%)	2080 (56.70)	6391 (62.60)	<.001
BMI (kg/m^2^)	28.49 (24.94-33.59)	28.49 (24.87-33.09)	0.214
Admission type, n (%)			<.001
Elective	616 (16.80)	3392 (33.20)	
Emergency/Urgent	3053 (83.20)	6812 (66.80)	
Race, n (%)			<.001
White	2208 (60.18)	6862 (67.25)	
Asian	112 (3.05)	254 (2.49)	
Black	284 (7.74)	627 (6.14)	
Hispanic	117 (3.19)	327 (3.20)	
American Indian/Alaska Native	7 (0.19)	13 (0.13)	
Other/unkown	941 (25.60)	2121 (20.79)	
Marital status, n (%)			<.001
Married	1498 (40.83)	5015 (49.15)	
Divorced	229 (6.24)	713 (6.99)	
Single	925 (25.21)	2455 (24.06)	
Widowed	422 (11.50)	976 (9.56)	
Unknown	595 (16.22)	1045 (10.24)	
Service unit (MICU%)	1931 (52.60)	2326 (22.80)	<.001
Severity of illness			
SOFA score	4 (3-6)	3 (2-4)	<.001
SAPS II score	50 (39-62)	37 (30-46)	<.001
OASIS score	42 (36-49)	35 (30-42)	<.001
APS III score	76 (54.50-101)	47 (33-68)	<.001
LODS score	9 (6-12)	5 (3-8)	<.001
SIRS score	3 (3-4)	3 (2-3)	<.001
Interventions, n (%)			
RRT use	859 (23.40)	626 (6.10)	<.001
Mechanical ventilation use	3137 (85.50)	8180 (80.20)	<.001
Vasopressor use	2844 (77.50)	6846 (67.10)	<.001
Elective surgery	56 (1.50)	825 (8.10)	<.001
Comorbidities, (n%)			
Hypertension	783 (21.30)	3733 (36.60)	<.001
Diabetes	1091 (29.70)	2932 (28.70)	0.251
CPD	995 (27.10)	2724 (26.70)	0.619
Coronary	1153 (31.40)	4385 (43.00)	<.001
CHF	1075 (29.30)	2781 (27.30)	0.018
Cancer	649 (17.70)	1034 (10.10)	<.001
Liver disease	1021 (27.80)	1017 (10.00)	<.001
Renal disease	874 (23.80)	1620 (15.90)	<.001
Cerebrovascular disease	413 (11.30)	1584 (15.50)	<.001
Shock	1383 (37.70)	948 (9.30)	<.001
Vital signs			
MAP (mmHg)	55 (46-73.50)	58 (52-82.50)	<.001
Heart rate (bpm)	112 (97-128)	101 (90-116)	<.001
Temperature (℃)	36.39 (35.70-37.67)	36.39 (35.80-37.67)	0.146
Respiratory rate (bpm)	30 (25-34)	26 (23-31)	<.001

BMI, Body mass index; MICU, Medical intensive care unit; SOFA, Sequential organ failure assessment; SAPS II, Simplified acute physiology score II; OASIS, Oxford acute severity of illness score; APS III, Acute physiology score III; LODS, Logistic organ dysfunction system; SIRS, Systemic inflammatory response syndrome; RRT, Renal replacement therapy; CPD, Chronic pulmonary disease; CHF, Congestive heart failure; MAP, Mean arterial pressure.

### Correlations between SABG-3 and outcomes in patients with sepsis

Univariable and multivariable logistic regression analyses were conducted to evaluate the effect of SABG-3 on 28-day mortality. The SABG-3 risk score was independently associated with increased 28-day mortality (adjusted odds ratio: 1.559; 95% CI: 1.464–1.659; *P* < 0.001) (Table 2). As shown in Table 3, sensitivity analyses across all five estimation models confirmed that high-risk patients had higher 28-day mortality and high-risk status was an independent prognostic indicator for ICU patients with sepsis. For the PSM analysis, a 1:1 matching algorithm yielded 1,456 pairs of high-risk and low-risk patients, with no significant differences in baseline characteristics between the two groups (S3 Table and S2 Fig). The associations between SABG-3-derived risk status and 28-day mortality remained significant, as confirmed by subgroup analyses of the original cohort (S3 Fig). Secondary outcomes were then assessed using PSM with a 1:1 matching algorithm. Both vasopressor-free days and mechanical ventilation-free days were shorter in the high-risk group than in the low-risk group (Table 4).

**Table 2:** Impact of the SABG-3-derived risk score and other important variables on 28-day mortality in sepsis.

Variables	Univariable models		Full multivariable model	
	Odds ratio (95% CI)	*P* value	Odds ratio (95% CI)	*P* value
SABG-3-derived risk score	1.966 (1.885-2.050)	<.001	1.559 (1.464-1.659)	<.001
Age	1.018 (1.015-1.021)	<.001	1.040 (1.032-1.047)	<.001
Gender (Male)	0.784 (0.719-0.855)	<.001	0.881 (0.748-1.038)	0.131
BMI	0.982 (0.975-0.989)	<.001	0.970 (0.959-0.981)	<.001
Admission type	3.500 (3.092-3.962)	<.001	1.441 (1.169-1.776)	0.001
Race				
White	Reference		Reference	
Asian	1.054 (0.798-1.393)	0.709	0.609 (0.391-0.949)	0.029
Black	1.255 (1.056-1.493)	0.010	0.830 (0.629-1.094)	0.185
Hispanic	0.823 (0.625-1.085)	0.168	0.781 (0.504-1.212)	0.271
American Indian/Alaska Native	0.574 (0.133-2.478)	0.457	0.415 (0.052-3.323)	0.407
Other	0.945 (0.763-1.171)	0.604	0.815 (0.559-1.188)	0.287
Marital status				
Married	Reference		Reference	
Divorced	1.048 (0.870-1.263)	0.619	0.881 (0.651-1.194)	0.415
Single	1.038 (0.927-1.163)	0.518	1.037 (0.856-1.255)	0.712
Widowed	1.670 (1.451-1.923)	<.001	1.003 (0.786-1.281)	0.980
Service unit	2.842 (2.604-3.103)	<.001	1.430 (1.205-1.696)	<.001
Severity of illness				
SOFA score	1.183 (1.162-1.204)	<.001	0.965 (0.930-1.000)	0.052
SAPS II score	1.061 (1.058-1.064)	<.001	0.988 (0.981-0.996)	0.003
OASIS score	1.106 (1.100-1.112)	<.001	0.980 (0.966-0.993)	0.003
APS III score	1.040 (1.038-1.042)	<.001	1.020 (1.015-1.026)	<.001
LODS score	1.348 (1.329-1.366)	<.001	1.127 (1.081-1.175)	<.001
SIRS score	1.444 (1.370-1.522)	<.001	0.949 (0.854-1.054)	0.325
Interventions				
RRT use	3.826 (3.414-4.287)	<.001	1.243 (0.997-1.550)	0.053
Mechanical ventilation use	2.438 (2.122-2.800)	<.001	1.798 (1.314-2.459)	<.001
Vasopressor use	2.522 (2.258-2.817)	<.001	1.285 (1.043-1.585)	0.019
Elective surgery	0.089 (0.056-0.141)	<.001	0.275 (0.134-0.567)	<.001
Comorbidities				
Hypertension	0.594 (0.538-0.655)	<.001	0.830 (0.684-1.007)	0.059
Diabetes	0.957 (0.870-1.052)	0.360	0.973 (0.815-1.162)	0.766
CPD	1.131 (1.029-1.243)	0.011	1.081 (0.913-1.279)	0.366
Coronary	0.706 (0.645-0.772)	<.001	0.922 (0.772-1.102)	0.373
CHF	1.359 (1.240-1.490)	<.001	1.328 (1.112-1.585)	0.002
Cancer	2.266 (2.024-2.538)	<.001	2.081 (1.690-2.562)	<.001
Liver disease	2.725 (2.457-3.024)	<.001	1.590 (1.294-1.954)	<.001
Renal disease	1.513 (1.365-1.679)	<.001	0.963 (0.781-1.188)	0.727
Cerebrovascular disease	1.645 (1.473-1.838)	<.001	2.562 (2.101-3.125)	<.001
Shock	4.519 (4.099-4.982)	<.001	1.355 (1.121-1.638)	0.002
Vital signs				
MAP (mmHg)	0.992 (0.990-0.994)	<.001	1.002 (1.000-1.004)	0.073
Heart rate (bpm)	1.014 (1.012-1.016)	<.001	1.001 (0.998-1.005)	0.446
Temperature (℃)	0.872 (0.846-0.899)	<.001	0.973 (0.919-1.030)	0.348
Respiratory rate (bpm)	1.034 (1.030-1.039)	<.001	1.001 (0.992-1.009)	0.885

BMI, Body mass index; SOFA, Sequential organ failure assessment; SAPS II, Simplified acute physiology score II; OASIS, Oxford acute severity of illness score; APS III, Acute physiology score III; LODS, Logistic organ dysfunction system; SIRS, Systemic inflammatory response syndrome; RRT, Renal replacement therapy; CPD, Chronic pulmonary disease; CHF, Congestive heart failure; MAP, Mean arterial pressure.

**Table 3:** Primary outcome analyses across five models elucidating the role of SABG-3 in septic patients.

Methods	Odds ratio	Confidence interval		*P* value
		2.5% 97.5%		
Doubly robust with unbalanced covariates	2.820	2.404	3.307	<.001
Doubly robust with all covariates	2.807	2.384	3.305	<.001
Propensity score IPW	2.405	2.205	2.624	<.001
Propensity score matching	2.230	1.867	2.664	<.001
Multivariable after multiple imputations	2.814	2.511	3.155	<.001

IPW, Inverse probability weighting.

**Table 4:** Secondary outcome analyses with propensity score-matched cohorts.

Secondary outcomes	High risk (n = 1482)	Low risk (n = 1482)	*P* value
Vasopressor use	1140 (76.90%)	1107 (74.70%)	0.157
Vasopressor-free days	2.450 (0.634-5.526)	3.607 (1.203-7.420)	<.001
Mechanical ventilation use	1322 (89.20%)	1303 (87.90%)	0.273
Mechanical ventilation-free days	2.350 (0.673-5.000)	3.136 (1.171-6.652)	<.001

Collectively, these data indicate that SABG-3 is an effective prognostic tool for ICU patients with sepsis.

### Internal and external validation of SABG-3 in ICU patients with septic shock and sepsis

Internal validation of SABG-3 was then performed in patients with septic shock. Patients in the high-risk group exhibited lower survival than those in the low-risk group. ROC curve analysis yielded AUC values of 0.734, 0.711, and 0.706 for predicting 28-, 90-, and 180-day survival, respectively, in patients with septic shock (S4 Fig). Moreover, among patients with septic shock, those with high risk were associated with greater illness severity (S4 Table). Consistent with these results, univariable and multivariable logistic regression analyses revealed that high-risk patients with septic shock had a poor prognosis (adjusted odds ratio: 1.432; 95% CI: 1.321–1.552; *P* < 0.001) (S5 Table). In addition, sensitivity analyses across all five estimation models in patients with septic shock confirmed a positive association between high-risk status and 28-day mortality (S6 Table). Subsequently, external validation of SABG-3 was performed using the eICU-CRD. Patients with sepsis in the high-risk group exhibited lower survival rates than those in the low-risk group. ROC curve analysis yielded AUC values of SABG-3 of 0.698, 0.705, and 0.704 for predicting 28‑, 90‑, and 180‑day survival, respectively (S5 Fig). In addition, high-risk patients with sepsis exhibited greater illness severity, compared with low-risk patients (S7 Table). Univariable and multivariable logistic regression analyses confirmed that high‑risk patients with sepsis had a poor prognosis (S8 Table). Notably, SABG-3 demonstrated practical advantages over SOFA and SIRS, while maintaining performance comparable to other widely used scales, including SAPS II, OASIS, APS III, and LODS (S6 Fig). Collectively, these data confirm that SABG-3 is a reliable prognostic tool for predicting outcomes in ICU patients with septic shock and sepsis.

### Establishment of SABG-3-derived nomograms in patients with sepsis

**Fig 4:**
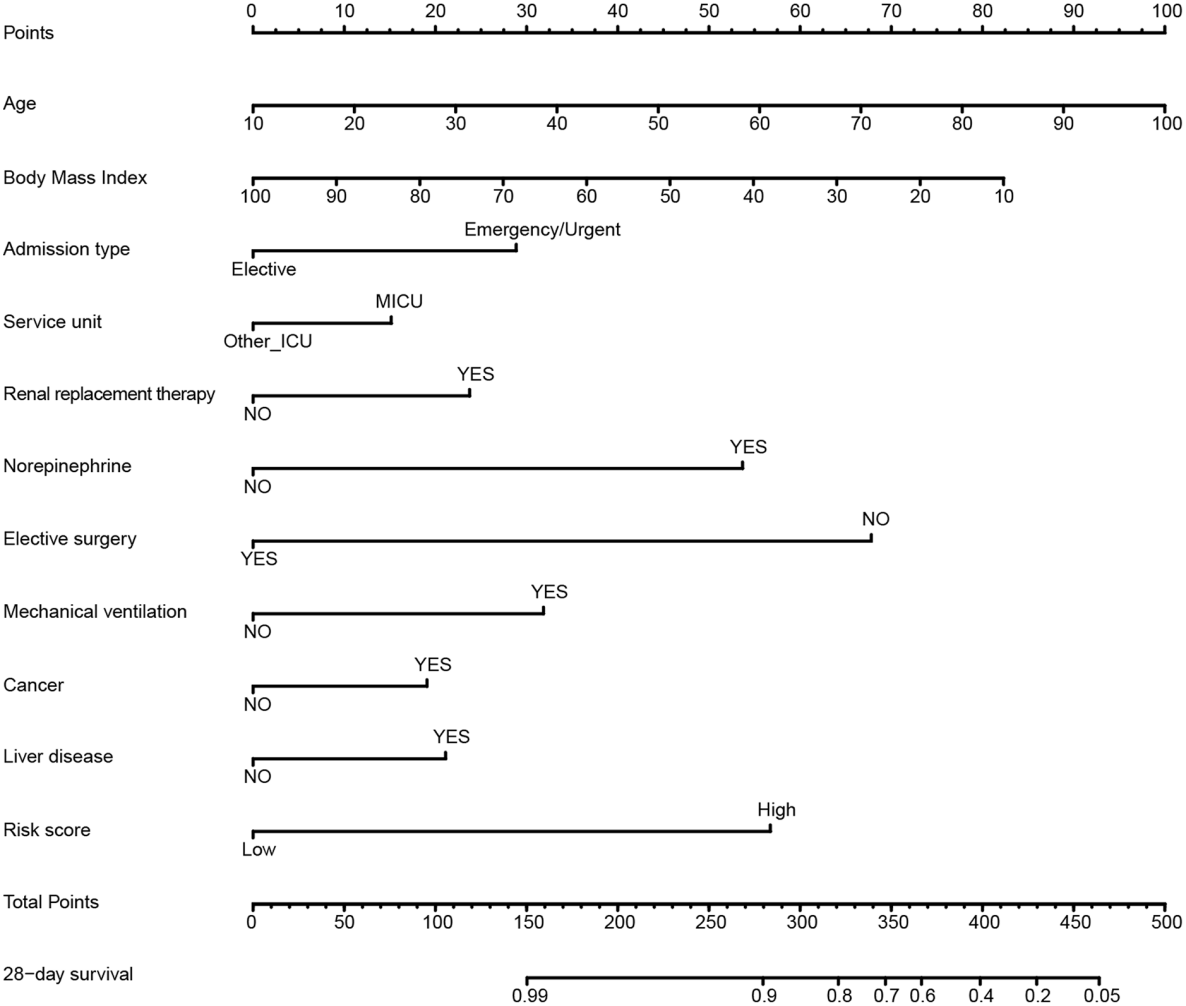
Construction of the SABG-3-derived nomogram for predicting survival in patients with sepsis. The nomogram included 11 variables: SABG-3 risk status, age, BMI, admission type, ICU unit type, renal replacement therapy, norepinephrine use, mechanical ventilation, elective surgery, cancer, and liver disease. SABG-3: Sepsis assessment blood gas 3.

After confirming the vital role of SABG-3 in prognostic evaluation, we developed prognostic nomograms by integrating risk status with other clinical variables to obtain an accurate and comprehensive predictive tool. Collinearity analysis, univariable logistic regression, LASSO regression, multivariable logistic regression, and doubly robust analyses were employed to ensure robust variable selection. Ultimately, 11 variables were incorporated into the nomogram: SABG-3-determined risk, age, BMI, admission type, service unit, use of renal replacement therapy, norepinephrine use, mechanical ventilation use, elective surgery, cancer status, and liver disease status (Fig 4). The calibration plot demonstrated high agreement between predicted and observed risks, confirming the accuracy of the risk estimates of the model (S7 Fig). Additional information on the doubly robust model is summarized in S9 Table. Furthermore, the C-index, F1 score, and accuracy of the constructed nomogram outperformed established clinical scales (Table 5), indicating excellent performance in predicting the prognosis of patients with sepsis. Moreover, a comparative analysis of the established nomogram and widely used scales was performed to assess their complexity (Table 6), confirming that the SABG-3-derived nomogram was more convenient to apply than existing prognostic scales.

**Table 5:** C-indexes, F1 score, and accuracy of the established nomogram and widely used scales.

Models	C-indexes/AUC (95% Confidence interval)	F1 score	Accuracy (%)
SABG-3-derived nomogram	0.850 (0.841-0.860)	0.526	77.7
SABG-3	0.768 (0.756-0.780)	0.495	77.20
SOFA	0.604 (0.592-0.617)	0.345	57.82
SAPS II	0.748 (0.738-0.759)	0.456	70.59
OASIS	0.745 (0.734-0.755)	0.444	67.33
APS III	0.802 (0.793-0.811)	0.493	70.22
LODS	0.784 (0.774-0.793)	0.482	70.70
SIRS	0.582 (0.570-0.594)	0.303	67.66

SABG-3, Sepsis assessment blood gas 3; SOFA, Sequential organ failure assessment; SAPS II, Simplified acute physiology score II; OASIS, Oxford acute severity of illness score; APS III, Acute physiology score III; LODS, Logistic organ dysfunction system; SIRS, Systemic inflammatory response syndrome.

**Table 6:** Comparison analysis of the established nomogram and widely used scales.

Models	Source of variables	Number of variables	Estimated time for variable acquisition
SABG-3-derived nomogram	Blood gas tests; patients’ medical records; circulatory and respiratory support	11	30 min
SABG-3	Blood gas tests	3	15 min
SOFA	Blood gas tests; blood routine tests; biochemical tests; circulatory and respiratory support; Glasgow coma scale; urine output	10	24 h
SAPS II	Blood gas tests; patients’ medical records; blood routine tests; biochemical tests; respiratory support; Glasgow coma scale; urine output	17	8 h
OASIS	Patients’ medical records; respiratory support; Glasgow coma scale; urine output	10	24 h
APS III	Blood gas tests; patients’ medical records; blood routine tests; biochemical tests; Glasgow coma scale	35	2 h
LODS	Blood gas tests; patients’ medical records; blood routine tests; biochemical tests; Glasgow coma scale; urine output; coagulation profile	13	24 h
SIRS	Patients’ medical records; blood routine tests	4	30 min

SABG-3, Sepsis assessment blood gas 3; SOFA, Sequential organ failure assessment; SAPS II, Simplified acute physiology score II; OASIS, Oxford acute severity of illness score; APS III, Acute physiology score III; LODS, Logistic organ dysfunction system; SIRS, Systemic inflammatory response syndrome.

## Discussion

The current international guideline for sepsis [21] use various blood gas analysis variables, including PO_2_, FiO_2_, and lactate levels, to support diagnosis. However, these guidelines did not recommend the use of arterial or venous lactate for diagnosing septic shock. In the present study, we demonstrated that the use of either arterial or venous serum lactate is clinically acceptable for diagnostic purposes. More importantly, we developed and validated a novel prognostic model-SABG-3-incorporating lactate, PO_2_, and BE from arterial blood gas analyses of patients with sepsis. The findings, supported by both internal and external validation, confirm the robust performance of this model in predicting the prognosis of patients with sepsis and septic shock.

A close association of blood gas parameters with outcomes has been reported in patients with sepsis [22]. In the current study, we developed a three-variable-based risk model (i.e., SABG-3) that demonstrated good prognostic performance in ICU patients with sepsis. Moreover, incorporating all blood gas parameters into the model yielded only marginal improvements despite a substantial increase in the number of variables. Therefore, we used the mean arterial blood gas values for further analyses. All three variables (i.e., PO_2_, BE, and lactate) were demonstrated to be related to sepsis outcomes [23,24]. These findings highlight the need to reconsider the optimal blood gas parameters for prognostic assessment in patients with sepsis. Using available models [15,25] (logistic regression, multivariable regression after multiple imputations, PSM, propensity score IPW, doubly robust analyses, and subgroup analyses), we confirmed SABG-3 as an independent prognostic indicator for both sepsis and septic shock after adjusting for multiple clinical characteristics. SABG-3 effectively stratified patients by their risk of requiring mechanical ventilation and vasopressors, suggesting its potential utility in identifying patients with sepsis benefiting from intensified therapy before disease progression. The rapid availability of mean blood gas values in SABG-3 facilitates the use of SABG-3 as a simple assessment tool for improved predicting outcomes in patients with sepsis. To address the complexity of existing prognostic scales in sepsis [26–28], we established an accurate and convenient nomogram by integrating the SABG-3 risk score with other clinical features. This SABG-3-derived nomogram demonstrated favorable discriminative performance (AUC: 0.850) that compares favorably with the model by Liu et al. (AUC: 0.59–0.80) [29] and the SOFA component score model by Pan et al. (AUC: 0.66–0.76) [30]. Logistic regression demonstrated predictive performance comparable to that of the machine learning techniques evaluated. Its performance was also comparable to that of the model developed by Hu et al. (AUC: 0.651–0.884) [31]. These results align with previous evidence suggesting that logistic regression can perform as effectively as complex machine learning algorithms in predicting adverse clinical outcomes and disease severity among critically ill patients [32]. Therefore, we conducted rigorous analyses, including logistic regression, to optimize the SABG-3-derived nomogram. Although the improvement of the SABG-3-derived nomogram over existing prognostic scales was modest, the nomogram comprises only 11 variables readily accessible from medical records. The variables required for the established nomogram are more rapidly accessible than those used by traditional scoring systems. Prior analyses suggest that an AUC increase of ≥0.03 represents a clinically and statistically meaningful improvement [33]. Our model exceeded this threshold with a minimum difference (SABG-3-derived nomogram vs APS III: 0.048). Moreover, the F1 score and accuracy of the SABG-3-derived nomogram outperformed existing scales, reinforcing the clinical significance of these results. Future development of a user-friendly interface could further enhance the practical utility of the nomogram. Thus, we propose that the SABG-3 model can be used for rapid and preliminary screening, and the derived nomogram for a more precise, detailed evaluation of patients with sepsis and septic shock.

This study has several notable strengths. First, it represents the first effort to develop and validate a prognostic risk model (SABG-3) based exclusively on routine blood gas parameters in sepsis. Second, the reliance on routinely available clinical variables enables rapid, widespread application in clinical settings. Third, the large-scale datasets derived from MIMIC‑IV and eICU-CRD ensured the reliability and statistical power of the results. Through a stepwise validation process, we developed the model in a broad sepsis cohort, internally validating it in the more severe septic shock subgroup, and externally validating it in an independent sepsis cohort. The model remained an independent predictor across all populations, demonstrating generalizability and providing clinical value for early risk stratification in patients at the highest risk of mortality.

Despite its strengths, this study has several limitations. First, the use of the MIMIC-IV and eICU-CRD databases may introduce inherent selection and information biases; furthermore, the lack of resampling-based cross-validation could increase overfitting risk. Second, hospital-level heterogeneity was not modeled, leaving the effect of site variation on model performance unaddressed; in addition, causal relationships could not be fully established despite confounder adjustment. Third, the conventional 20% missingness cutoff for variable exclusion, while reducing bias from imprecise imputation, was arbitrary and may discard potentially valuable prognostic data [34]. Finally, all continuous predictors were modeled linearly; without testing for nonlinear relationships, the model fit and predictive performance may be constrained. Consequently, multicenter prospective studies using standardized data collection and comprehensive confounder assessment are necessary to verify the prognostic value of SABG-3, establish causality, and enhance its generalizability and clinical utility.

## Conclusions

Real-world data from the MIMIC-IV and eICU-CRD can adequately assess the clinical effectiveness of diagnostic tests or interventions. In conclusion, this study developed and validated SABG-3 as a prognostic model based on blood gas parameters, for patients with sepsis and septic shock. Cross-validation clarified the links between blood gas parameters and prognosis in patients with sepsis. Moreover, the assessment model and its derived nomogram serve as a promising decision-support tool for clinicians, particularly for identifying high-risk patients who may benefit from intensified treatment, potentially improving clinical outcomes.

## Supporting information

S1 Table Three parameters of the prognostic model in the sepsis cohort. (PDF)

S2 Table Area under the curves of the established models. (PDF)

S3 Table Baseline characteristics of patients with sepsis between high-risk and low-risk groups after PSM. (PDF)

S4 Table Baseline characteristics of patients with septic shock between high-risk and low-risk groups. (PDF)

S5 Table Impact of risk score and other important variables on 28-day mortality in septic shock. (PDF)

S6 Table Primary outcome analyses across five estimation models in patients with septic shock. (PDF)

S7 Table Baseline characteristics of patients with sepsis in the eICU-CRD between high-risk and low-risk groups. (PDF)

S8 Table Impact of the SABG-3-derived risk score and other important variables on 28-day mortality in the eICU-CRD. (PDF)

S9 Table Details of the doubly robust model evaluating the effect of variables on 28-day mortality. (PDF)

S1 Fig Prognostic performance of serum lactate in patients with sepsis. Time-dependent receiver operating characteristic curves for arterial (upper) and venous (below) serum lactate levels.
(PDF)

S2 Fig Balanced distribution of baseline characteristics between high-risk and low-risk groups after propensity score matching. Propensity score distribution or absolute standardized mean differences confirm negligible variance between the control (low-risk) and treatment (high-risk) groups after propensity score matching.
(PDF)

S3 Fig Subgroup analyses of the association between SABG-3 and 28-day mortality in patients with sepsis. Subgroup analyses confirm the established model as an independent prognostic indicator. SABG-3: Sepsis assessment blood gas 3.
(PDF)

S4 Fig Prognostic role of the blood gas parameter–based assessment model in patients with septic shock. (**A**) Time-dependent receiver operating characteristic curves evaluating model performance. (**B**) Kaplan–Meier survival curves for high-risk or low-risk patients with septic shock (log-rank test) as determined by the blood gas parameter–based assessment model.
(PDF)

S5 Fig Prognostic role of the blood gas parameter–based assessment model in patients with sepsis from the eICU-CRD. (**A**) Time-dependent receiver operating characteristic curves of the established model for evaluating its performance in patients with sepsis. (**B**) Kaplan–Meier survival curves comparing high- or low-risk patients with sepsis as determined by the blood gas parameter–based assessment model.
(PDF)

S6 Fig Decision curve analysis of SABG-3. Decision curve analysis for comparing the net clinical benefit of SABG-3 with established sepsis severity scores. SABG-3: Sepsis assessment blood gas 3.
(PDF)

S7 Fig Calibration plot of the SABG-3-derived nomogram. Calibration diagram was plotted to evaluate the accuracy of the constructed nomogram. SABG-3: Sepsis assessment blood gas 3.
(PDF)

## Abbreviation:

AaDO_2_: alveolar-arterial oxygen gradient
APS III: acute physiology score III
AUC: area under the receiver operating characteristic curve
BE: base excess
BMI: body mass index
CIs: confidence intervals
eICU-CRD: electronic Intensive Care Unit Collaborative Research Database
FiO_2_: fraction of inspired oxygen
ICD: classification of diseases
ICU: intensive care unit
IPW: inverse probability weighting
LASSO: least absolute shrinkage and selection operator
LODS: logistic organ dysfunction system
MIMIC-IV: Medical Information Mart for Intensive Care IV
OASIS: Oxford acute severity of illness score
OI: oxygenation index
PCO_2_: partial pressure of carbon dioxid
PO_2_: partial pressure of oxygen
PSM: propensity score matching
ROC: receiver operating characteristic
SABG-3: sepsis assessment blood gas 3
SAPS II: simplified acute physiology score II
SIRS: systemic inflammatory response syndrome
SOFA: sequential organ failure assessment
