# Supplementary material for: A blood gas parameter–based assessment model for predicting poor prognosis in sepsis: A retrospective analysis of the MIMIC-IV and eICU-CRD
Source: PLoS One. 2026 Jul 9;21(7):e0346532. doi: 10.1371/journal.pone.0346532 (PMC13349094; doi:10.1371/journal.pone.0346532)
Supplement: S1 Table — (PDF) [file pone.0346532.s001.pdf]

S1 Table. Three parameters of the prognostic model in the sepsis cohort.

Variables	coefficients	OR	OR.95% lower	OR.95% upper	*P* value
PO_2_mean	-0.010205526	0.989846374	0.989047403	0.990645991	1.84E-135
BE_mean	-0.087313628	0.916389645	0.910515744	0.92230144	4.85E-156
lactate_mean	0.222091272	1.248685342	1.23368043	1.263872755	6.70E-284

OR: Odds ratio
