# Supplementary material for: A blood gas parameter–based assessment model for predicting poor prognosis in sepsis: A retrospective analysis of the MIMIC-IV and eICU-CRD
Source: PLoS One. 2026 Jul 9;21(7):e0346532. doi: 10.1371/journal.pone.0346532 (PMC13349094; doi:10.1371/journal.pone.0346532)
Supplement: S2 Table — (PDF) [file pone.0346532.s002.pdf]

S2 Table. Area under the curves of the established models.

Blood gas parameters-based models	Area under curves
Mean values of arterial (during the first day)	0.768
Initial values of arterial (during the first day)	0.761
Maximum values of arterial (during the first day)	0.701
Minimum values of arterial (during the first day)	0.672
Arterial difference (during the first day)	0.720
Mean values of venous (during the first day)	0.680
Initial values of venous (during the first day)	0.673
Maximum values of venous (during the first day)	0.692
Minimum values of venous (during the first day)	0.670
Venous difference (during the first day)	0.570
