# Supplementary material for: A blood gas parameter–based assessment model for predicting poor prognosis in sepsis: A retrospective analysis of the MIMIC-IV and eICU-CRD
Source: PLoS One. 2026 Jul 9;21(7):e0346532. doi: 10.1371/journal.pone.0346532 (PMC13349094; doi:10.1371/journal.pone.0346532)
Supplement: S3 Table — (PDF) [file pone.0346532.s003.pdf]

S3 Table. Baseline characteristics of patients with sepsis between high-risk and low-risk groups after PSM.

Variables	After PSM(n=2912)	High group(n=1456)	Low group (n=1456)	*P* value
Age (years)				
Male, n (%)	1662 (57.10)	837 (57.50)	825 (56.70)	0.653
BMI (kg/m^2)	28.37 (24.46-33.58)	28.47 (24.70-33.63)	28.24 (24.31-33.53)	0.255
Admission type, n (%)				0.322
Elective	628 (21.60)	325 (22.30)	303 (20.80)	
Emergency/Urgent	2284 (78.40)	1131 (77.70)	1153 (79.20)	
Race, n (%)				0.512
White	2249 (77.20)	1115 (76.60)	1134 (77.90)	
Asian	108 (3.70)	54 (3.70)	54 (3.70)	
Black	284 (9.80)	154 (10.60)	130 (8.90)	
Hispanic	106 (3.60)	55 (3.80)	51 (3.50)	
American Indian / Alaska Native	9 (0.30)	6 (0.40)	3 (0.20)	
Other	156 (5.40)	72 (4.90)	84 (5.80)	
Marital status, n (%)				0.796
Married	1441 (49.50)	716 (49.20)	725 (49.80)	
Divorced	239 (8.20)	121 (8.30)	118 (8.10)	
Single	865 (29.70)	427 (29.30)	438 (30.10)	
Widowed	367 (12.60)	192 (13.20)	175 (12.00)	
Service unit (MICU%)	1314 (45.10)	654 (44.90)	660 (45.30)	0.823
Severity of illness				
SOFA score	4 (3-6)	4 (3-6)	4 (2-6)	0.262
SAPS II score	46 (37-56)	46 (37-56)	46 (36-56)	0.631
OASIS score	41 (35-47)	41 (35-47)	41 (35-47)	0.658
APS III score	69 (51-90)	69 (51-90)	70 (51-90)	0.732
LODS score	8 (6-10)	8 (6-10)	8 (6-11)	0.991
SIRS score	3 (3-4)	3 (3-4)	3 (3-4)	0.448
Interventions, n (%)				
RRT use	533 (18.30)	271 (18.60)	262 (18.00)	0.666
Mechanical ventilation use	2567 (88.20)	1281 (88.00)	1286 (88.30)	0.774
Vasopressor use	2220 (76.20)	1115 (76.60)	1105 (75.90)	0.663
Elective surgery	73 (2.50)	36 (2.50)	37 (2.50)	0.906
Comorbidities, (n%)				
Hypertension	651 (22.40)	331 (22.70)	320 (22.00)	0.625
Diabetes	905 (31.10)	463 (31.80)	442 (30.40)	0.400
CPD	808 (27.70)	400 (27.50)	408 (28.00)	0.741
Coronary	983 (33.80)	491 (33.70)	492 (33.80)	0.969
CHF	899 (30.90)	456 (31.30)	443 (30.40)	0.602
Cancer	507 (17.40)	250 (17.20)	257 (17.70)	0.732
Liver disease	687 (23.60)	339 (23.30)	348 (23.90)	0.694
Renal disease	731 (25.10)	369 (25.30)	362 (24.90)	0.765
Cerebrovascular disease	341 (11.70)	171 (11.70)	170 (11.70)	0.954
Shock	873 (30.00)	435 (29.90)	438 (30.10)	0.903
Vital signs				
MAP (mmHg)	56 (49-78)	56 (48-78)	56 (49.5-78.5)	0.246
Heart rate (bpm)	109 (95-125)	110 (95-125)	109 (95-125)	0.541
Temperature (°C)	36.39 (35.83-37.72)	36.39 (35.83-37.69)	36.39 (35.83-37.72)	0.969
Respiratory rate (bpm)	29 (24-34)	29 (25-34)	28 (24-33)	0.092

PSM: Propensity score matching

BMI: Body mass index

MICU: Medical intensive care unit

SOFA: Sequential organ failure assessment

SAPS II: Simplified acute physiology score II

OASIS: Oxford acute severity of illness score
APS III: Acute physiology score III
LODS: Logistic organ dysfunction system
SIRS: Systemic inflammatory response syndrome
RRT: Renal replacement therapy
CPD: Chronic pulmonary disease
CHF: Congestive heart failure
MAP: Mean arterial pressure
