# Supplementary material for: A blood gas parameter–based assessment model for predicting poor prognosis in sepsis: A retrospective analysis of the MIMIC-IV and eICU-CRD
Source: PLoS One. 2026 Jul 9;21(7):e0346532. doi: 10.1371/journal.pone.0346532 (PMC13349094; doi:10.1371/journal.pone.0346532)
Supplement: S4 Table — (PDF) [file pone.0346532.s004.pdf]

S4 Table. Baseline characteristics of patients with septic shock between high-risk and low-risk groups.

Variables	High-risk (n=1759)	Low-risk (n=1044)	*P* value
Age (years)	67 (56-78)	66 (54-77)	0.016
Male, n (%)	951 (54.10)	615 (58.90)	0.013
BMI (kg/m^2)	28.31 (24.59-33.81)	28.52 (23.88-34.91)	0.955
Admission type, n (%)			0.413
Elective	222 (12.60)	143 (13.70)	
Emergency/Urgent	1537 (87.40)	901 (86.30)	
Race, n (%)			0.359
White	1107 (62.93)	662 (63.41)	
Asian	53 (3.01)	30 (2.87)	
Black	168 (9.55)	72 (6.90)	
Hispanic	51 (2.90)	26 (2.49)	
American Indian / Alaska Native	3 (0.17)	2 (0.19)	
Other / unkown	377 (21.43)	252 (24.14)	
Marital status, n (%)			0.010
Married	692 (39.34)	413 (39.56)	
Divorced	130 (7.39)	77 (7.38)	
Single	465 (26.44)	318 (30.46)	
Widowed	247 (14.04)	107 (10.25)	
Unknown	225 (12.79)	129 (12.36)	
Service unit (MICU%)	1194 (67.90)	574 (55)	<.001
Severity of illness			
SOFA score	4 (3-7)	4 (3-6)	<.001
SAPS II score	53 (43-65)	44 (37-54)	<.001
OASIS score	44 (36-50)	42 (36-48)	<.001
APS III score	84 (64-108)	69 (53-90)	<.001
LODS score	10 (7-12)	8 (6-11)	<.001
SIRS score	3 (3-4)	3 (3-4)	0.037
Interventions, n (%)			
RRT use	474 (26.90)	187 (17.90)	<.001
Mechanical ventilation use	1283 (72.90)	890 (85.20)	<.001
Vasopressor use	1589 (90.30)	941 (90.10)	0.862
Elective surgery	7 (0.40)	6 (0.60)	0.505
Comorbidities, (n%)			
Hypertension	341 (19.40)	272 (26.10)	<.001
Diabetes	571 (32.50)	338 (32.40)	0.962
CPD	473 (26.90)	319 (30.60)	0.037
Coronary	500 (28.40)	348 (33.30)	0.006
CHF	533 (30.30)	397 (38.00)	<.001
Cancer	361 (20.50)	155 (14.80)	<.001
Liver disease	550 (31.30)	188 (18.00)	<.001
Renal disease	450 (25.60)	248 (23.80)	0.279
Cerebrovascular disease	175 (9.90)	124 (11.90)	0.110
Vital signs			
MAP (mmHg)	52 (43-59)	55 (48-61)	<.001
Heart rate (bpm)	116 (101-132)	112 (97.75-128)	<.001
Temperature (℃)	36.64 (35.89-37.78)	37.17 (36.11-38.17)	<.001
Respiratory rate (bpm)	31 (26-35)	29 (25-33)	<.001

BMI: Body mass index

MICU: Medical intensive care unit

SOFA: Sequential organ failure assessment

SAPS II: Simplified acute physiology score II

OASIS: Oxford acute severity of illness score
APS III: Acute physiology score III
LODS: Logistic organ dysfunction system
SIRS: Systemic inflammatory response syndrome
RRT: Renal replacement therapy
CPD: Chronic pulmonary disease
CHF: Congestive heart failure
MAP: Mean arterial pressure
