# Supplementary material for: A blood gas parameter–based assessment model for predicting poor prognosis in sepsis: A retrospective analysis of the MIMIC-IV and eICU-CRD
Source: PLoS One. 2026 Jul 9;21(7):e0346532. doi: 10.1371/journal.pone.0346532 (PMC13349094; doi:10.1371/journal.pone.0346532)
Supplement: S5 Table — (PDF) [file pone.0346532.s005.pdf]

S5 Table. Impact of risk score and other important variables on 28-day mortality in septic shock.

Variables	Univariable models		Full multivariable model	
	**Odds ratio (95% CI)**	***P* value**	**Odds ratio (95% CI)**	***P* value**
SABG-3-derived risk score	1.426 (1.359-1.497)	<.001	1.432 (1.321-1.552)	<.001
Age	1.020 (1.015-1.026)	<.001	1.038 (1.025-1.050)	<.001
Gender (Male)	1.015 (0.871-1.183)	0.852	0.774 (0.586-1.021)	0.070
BMI	0.994 (0.984-1.004)	0.222	0.990 (0.973-1.006)	0.226
Admission type	1.262 (1.002-1.591)	0.048	1.099 (0.744-1.623)	0.635
Race				
White	Reference		Reference	
Asian	0.986 (0.624-1.560)	0.953	0.517 (0.222-1.204)	0.126
Black	1.009 (0.763-1.335)	0.951	0.884 (0.569-1.373)	0.583
Hispanic	0.941 (0.583-1.518)	0.803	1.189 (0.583-2.426)	0.634
American Indian/Alaska Native	0	0.999	0	0.999
Other	0.976 (0.661-1.441)	0.902	1.097 (0.592-2.033)	0.769
Marital status				
Married	Reference		Reference	
Divorced	0.769 (0.563-1.051)	0.100	0.862 (0.526-1.415)	0.558
Single	0.694 (0.572-0.841)	<.001	0.792 (0.578-1.086)	0.148
Widowed	1.069 (0.838-1.364)	0.590	0.691 (0.458-1.044)	0.079
Service unit	1.155 (0.986-1.354)	0.074	1.103 (0.840-1.449)	0.482
Severity of illness				
SOFA score	1.119 (1.087-1.151)	<.001	0.978 (0.927-1.033)	0.434
SAPS II score	1.055 (1.049-1.061)	<.001	0.992 (0.979-1.004)	0.191
OASIS score	1.070 (1.061-1.080)	<.001	0.975 (0.953-0.997)	0.029
APS III score	1.031 (1.028-1.034)	<.001	1.014 (1.006-1.023)	0.001
LODS score	1.254 (1.224-1.286)	<.001	1.116 (1.041-1.196)	0.002
SIRS score	1.213 (1.098-1.340)	<.001	0.926 (0.770-1.113)	0.414
Interventions				
RRT use	2.073 (1.737-2.473)	<.001	1.313 (0.949-1.817)	0.100
Mechanical ventilation use	2.086 (1.713-2.541)	<.001	2.462 (1.505-4.027)	<.001
Vasopressor use	2.359 (1.755-3.171)	<.001	0.784 (0.462-1.331)	0.367
Elective surgery	0.704 (0.216-2.291)	0.560	0	0.834
Comorbidities				
Hypertension	0.915 (0.760-1.101)	0.345	1.019 (0.718-1.446)	0.918
Diabetes	0.930 (0.790-1.095)	0.383	0.976 (0.729-1.307)	0.871
CPD	1.074 (0.908-1.271)	0.403	1.286 (0.968-1.708)	0.083
Coronary	1.121 (0.950-1.321)	0.175	1.196 (0.881-1.623)	0.251
CHF	1.073 (0.913-1.260)	0.394	1.194 (0.884-1.613)	0.247
Cancer	1.838 (1.516-2.229)	<.001	2.044 (1.446-2.890)	<.001
Liver disease	2.052 (1.730-2.434)	<.001	1.734 (1.260-2.386)	0.001
Renal disease	1.211 (1.018-1.442)	0.031	1.147 (0.822-1.600)	0.421
Cerebrovascular disease	1.194 (0.936-1.522)	0.154	2.103 (1.396-3.167)	<.001
Vital signs				
MAP (mmHg)	0.993 (0.990-0.995)	<.001	0.999 (0.996-1.003)	0.772
Heart rate (bpm)	1.004 (1.001-1.007)	0.010	1.003 (0.997-1.009)	0.362
Temperature (°C)	0.774 (0.735-0.816)	<.001	0.913 (0.830-1.003)	0.058
Respiratory rate (bpm)	1.012 (1.004-1.021)	0.003	1.004 (0.989-1.018)	0.621

BMI: Body mass index

SOFA: Sequential organ failure assessment

SAPS II: Simplified acute physiology score II

OASIS: Oxford acute severity of illness score

APS III: Acute physiology score III

LODS: Logistic organ dysfunction system
SIRS: Systemic inflammatory response syndrome
RRT: Renal replacement therapy
CPD: Chronic pulmonary disease
CHF: Congestive heart failure
MAP: Mean arterial pressure
