# Supplementary material for: A blood gas parameter–based assessment model for predicting poor prognosis in sepsis: A retrospective analysis of the MIMIC-IV and eICU-CRD
Source: PLoS One. 2026 Jul 9;21(7):e0346532. doi: 10.1371/journal.pone.0346532 (PMC13349094; doi:10.1371/journal.pone.0346532)
Supplement: S6 Table — (PDF) [file pone.0346532.s006.pdf]

S6 Table. Primary outcome analyses across five estimation models in patients with septic shock.

Methods	Odds ratio	Confidence interval		*P* value
		2.5%	97.5%	
Doubly robust with unbalanced covariates	2.453	1.958	3.072	<.001
Doubly robust with all covariates	2.358	1.792	3.101	<.001
Propensity score IPW	1.941	1.668	2.261	<.001
Propensity score matching	1.867	1.389	2.511	<.001
Multivariable after multiple imputations	2.503	2.041	3.070	<.001

IPW: Inverse probability weighting
