# Supplementary material for: A blood gas parameter–based assessment model for predicting poor prognosis in sepsis: A retrospective analysis of the MIMIC-IV and eICU-CRD
Source: PLoS One. 2026 Jul 9;21(7):e0346532. doi: 10.1371/journal.pone.0346532 (PMC13349094; doi:10.1371/journal.pone.0346532)
Supplement: S7 Table — (PDF) [file pone.0346532.s007.pdf]

S7 Table. Baseline characteristics of patients with sepsis in the eICU-CRD between high-risk and low-risk groups.

Variables	High-risk (n=2831)	Low-risk (n=1011)	*P* value
Age (years)	66 (55-76)	67 (55-77)	0.126
Male, n (%)	1532 (54.10)	507 (50.10)	0.03
BMI (kg/m^2)	27.98 (23.18-34.36)	26.91 (22.65-32.71)	<.001
Admission type, n (%)			0.593
Elective	1241 (43.80)	453 (44.80)	
Emergency/Urgent	1590 (56.20)	558 (55.20)	
Race, n (%)			0.140
White	2309 (81.60)	793 (78.40)	
Asian	55 (1.90)	27 (2.70)	
Black	246 (8.70)	108 (10.70)	
Hispanic	68 (2.40)	30 (3.00)	
Other / unkown	153 (5.40)	53 (5.20)	
Service unit (MICU%)	351 (12.40)	123 (12.20)	0.847
Severity of illness			
SOFA score	7 (4-10)	11 (7-14)	<.001
SAPS II score	42 (32-54)	59 (47-71.50)	<.001
OASIS score	33 (26-41)	41 (33-48)	<.001
APS III score	59 (41-78)	86 (63-113)	<.001
LODS score	6 (4-9)	10 (7-13)	<.001
SIRS score	1 (0-1)	1 (1-2)	<.001
Interventions, n (%)			
RRT use	234 (8.30)	182 (18.00)	<.001
Mechanical ventilation use	1389 (49.10)	658 (65.10)	<.001
Vasopressor use	1158 (40.90)	563 (55.70)	<.001
Elective surgery	108 (3.80)	46 (4.50)	0.306
Comorbidities, (n%)			
Hypertension	280 (9.90)	56 (5.50)	<.001
Diabetes	226 (8.00)	67 (6.60)	0.163
CPD	236 (8.30)	35 (3.50)	<.001
Coronary	59 (2.10)	19 (1.90)	0.692
CHF	74 (2.60)	20 (2.00)	0.261
Cancer	146 (5.20)	59 (5.80)	0.410
Liver disease	97 (3.40)	101 (10.00)	<.001
Renal disease	253 (8.90)	78 (7.70)	0.235
Cerebrovascular disease	50 (1.80)	19 (1.90)	0.816
Shock	516 (18.20)	323 (31.90)	<.001
Vital signs			
MAP (mmHg)	79.5 (67-97)	72 (61-86)	0.007
Heart rate (bpm)	98 (84-114)	106 (89-119)	<.001
Temperature (℃)	36.9 (36.40-37.70)	36.6 (36.00-37.30)	<.001
Respiratory rate (bpm)	22 (17-27)	23 (19-29)	<.001

BMI: Body mass index

MICU: Medical intensive care unit

SOFA: Sequential organ failure assessment

SAPS II: Simplified acute physiology score II

OASIS: Oxford acute severity of illness score

APS III: Acute physiology score III

LODS: Logistic organ dysfunction system

SIRS: Systemic inflammatory response syndrome

RRT: Renal replacement therapy
CPD: Chronic pulmonary disease
CHF: Congestive heart failure
MAP: Mean arterial pressure

S8 Table. Impact of the SABG-3-derived risk score and other important variables on 28-day mortality in patients with sepsis from the eICU-CRD.

Variables	Univariable models		Full multivariable model	
	Odds ratio (95% CI)	*P* value	Odds ratio (95% CI)	*P* value
SABG-3-derived risk score	21.651 (15.815-29.641)	<.001	301.886 (2.166-42082.601)	0.023
Age	0.984 (0.980-0.989)	<.001	1.018 (0.935-1.108)	0.682
Gender (Male)	0.910 (0.796-1.040)	0.167	0.194 (0.027-1.400)	0.104
BMI	1.000 (1.000-1.000)	0.782	0.988 (0.877-1.114)	0.846
Admission type	1.343 (1.175-1.535)	<.001	2.351 (0.184-30.107)	0.511
Race				
White	Reference		Reference	

Asian	0.969 (0.719-1.306)	0.835	0.194 (0.001-68.570)	0.584
Black	0.873 (0.512-1.490)	0.618	0.085 (0.000-76.765)	0.478
Hispanic	0.827 (0.577-1.186)	0.301	0.013 (0.000-11.419)	0.210
Other	1.142 (0.680-1.916)	0.616	/	1.000
Service unit	0.903 (0.739-1.103)	0.319	0.009 (0.000-14.899)	0.214
Severity of illness				
SOFA score	0.826 (0.811-0.842)	<.001	0.841 (0.534-1.324)	0.454
SAPS II score	0.948 (0.943-0.952)	<.001	0.992 (0.854-1.152)	0.917
OASIS score	0.945 (0.938-0.951)	<.001	0.992 (0.854-1.152)	0.917
APS III score	0.983 (0.981-0.985)	<.001	1.018 (0.983-1.054)	0.315
LODS score	0.812 (0.796-0.828)	<.001	1.208 (0.774-1.886)	0.406
SIRS score	0.622 (0.572-0.678)	<.001	0.661 (0.145-3.016)	0.593
Interventions				
RRT use	0.384 (0.312-0.471)	<.001	0.462 (0.018-11.643)	0.639
Mechanical ventilation use	0.386 (0.336-0.444)	<.001	0.046 (0.003-0.786)	0.034
Vasopressor use	0.499 (0.436-0.571)	<.001	17.539 (1.599-192.401)	0.019
Elective surgery	0.973 (0.694-1.364)	0.872	0.128 (0.007-2.314)	0.164
Comorbidities				
Hypertension	1.266 (0.992-1.616)	0.058	0.354 (0.018-6.975)	0.495
Diabetes	1.223 (0.945-1.584)	0.126	1.326 (0.020-89.999)	0.896
CPD	0.972 (0.750-1.259)	0.831	0.233 (0.007-8.204)	0.422
Coronary	0.938 (0.588-1.497)	0.787	/	0.998
CHF	0.844 (0.554-1.285)	0.429	/	/
Cancer	0.656 (0.494-0.872)	0.004	1.369 (0.28-67.000)	0.874
Liver disease	0.445 (0.334-0.594)	<.001	23.199 (0.462-1166.077)	0.116
Renal disease	0.623 (0.496-0.782)	<.001	/	0.998
Cerebrovascular disease	0.475 (0.295-0.766)	0.002	/	1.000
Shock	0.579 (0.495-0.677)	<.001	0.617 (0.075-5.078)	0.653
Vital signs				
MAP (mmHg)	1.011 (0.999-1.023)	0.067	1.057 (0.999-1.118)	0.055
Heart rate (bpm)	0.999 (0.995-1.002)	0.480	0.995 (0.937-1.057)	0.880
Temperature (℃)	1.123 (1.026-1.228)	0.011	1.186 (0.469-3.002)	0.718
Respiratory rate (bpm)	0.986 (0.975-0.996)	0.009	1.050 (0.908-1.215)	0.512

BMI: Body mass index

SOFA: Sequential organ failure assessment

SAPS II: Simplified acute physiology score II

OASIS: Oxford acute severity of illness score

APS III: Acute physiology score III

LODS: Logistic organ dysfunction system

SIRS: Systemic inflammatory response syndrome

RRT: Renal replacement therapy

CPD: Chronic pulmonary disease
CHF: Congestive heart failure
MAP: Mean arterial pressure

S9 Table. Details of the doubly robust model evaluating the effect of variables on 28-day mortality.

Variables	Odds ratio (95% CI)	*P* value
Age	1.002 (1.002-1.003)	<.001
Body mass index	0.998 (0.997-0.998)	<.001
Admission type	1.583 (1.339-1.872)	<.001
Service unit	1.238 (1.087-1.411)	0.001
Renal replacement therapy	1.254 (1.058-1.487)	0.009
Norepinephrine	1.737 (1.496-2.017)	<.001
Elective surgery	0.319 (0.171-0.592)	<.001
Mechanical ventilation	1.628 (1.221-2.171)	0.001
Cancer	1.544 (1.298-1.837)	<.001

Liver disease	1.340 (1.145-1.569)	<.001

Supplementary Figures and Figure legends

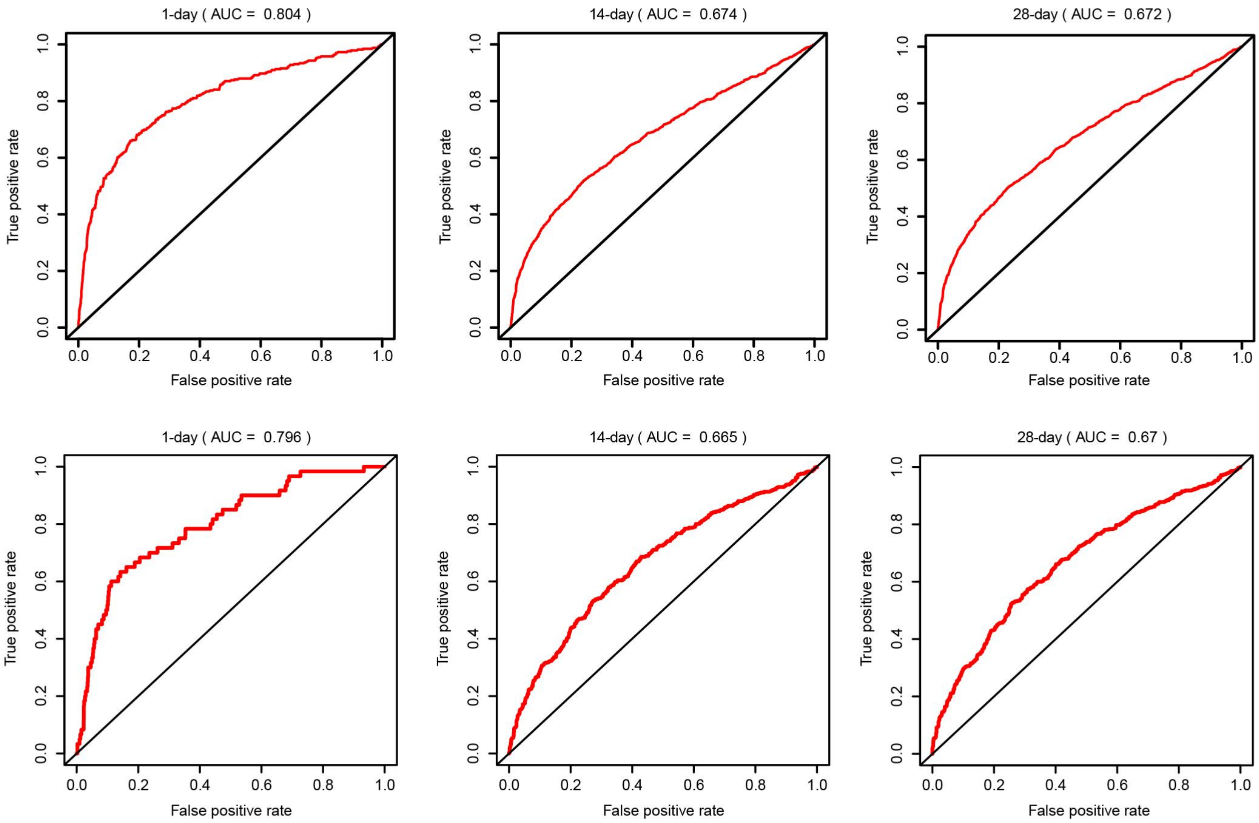

S1 Fig. Prognostic performance of serum lactate in patients with sepsis. Time-dependent receiver operating characteristic curves for arterial (upper) and venous (below) serum lactate levels.

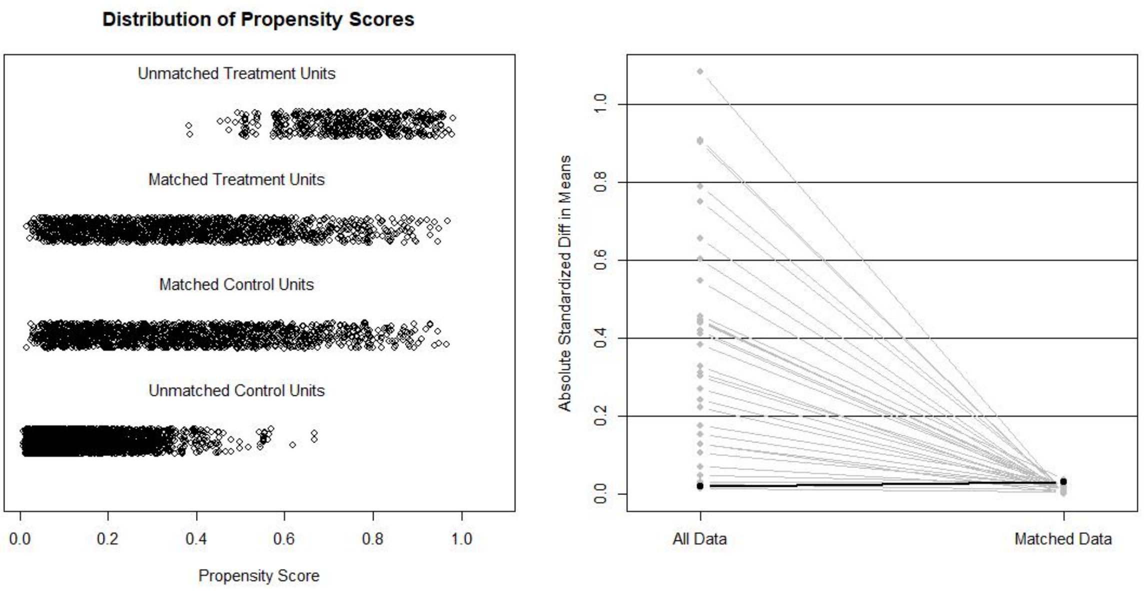

S2 Fig. Balanced distribution of baseline characteristics between high-risk and low-risk groups after propensity score matching. Propensity score distribution or absolute standardized mean differences confirm negligible variance between the control (low-risk) and treatment (high-risk) groups after propensity score matching.

Subgroup	High risk determined by SABG-3	No of patients	*P* value	Odds Ratio (95% CI)
**Gender**				
Female		5402	< 0.001	2.74 (2.23-3.38)
Male		8471	< 0.001	2.83 (2.36-3.38)
**Admission type**				
Elective		4008	< 0.001	2.65 (1.83-3.84)
Emergency/Urgent		9865	< 0.001	2.74 (2.37-3.17)
**Service unit**				
Medical intensive care unit		4257	< 0.001	2.23 (1.82-2.71)
Others		9616	< 0.001	3.25 (2.69-3.92)
**Renal replacement therapy**				
No		12388	< 0.001	2.53 (2.17-2.95)
Yes		1485	< 0.001	3.66 (2.71-4.96)
**Mechanical ventilation**				
No		2556	0.081	1.69 (0.94-3.04)
Yes		11317	< 0.001	2.78 (2.42-3.19)
**Vasopressor**				
No		4183	0.008	1.59 (1.13-2.25)
Yes		9690	< 0.001	3.08 (2.66-3.58)
**Elective surgery**				
No		12992	< 0.001	2.77 (2.42-3.17)
Yes		881	0.071	7.70 (0.84-70.39)
**Hypertension**				
No		9357	< 0.001	2.80 (2.40-3.26)
Yes		4516	< 0.001	2.75 (2.04-3.71)
**Congestive heart failure**				
No		10017	< 0.001	2.85 (2.40-3.38)
Yes		3856	< 0.001	2.65 (2.11-3.31)
**Cancer**				
No		12190	< 0.001	2.98 (2.57-3.47)
Yes		1683	< 0.001	2.07 (1.49-2.88)
**Liver disease**				
No		11835	< 0.001	2.70 (2.31-3.15)
Yes		2038	< 0.001	3.07 (2.30-4.09)
**Cerebrovascular disease**				
No		11876	< 0.001	2.87 (2.47-3.33)
Yes		1997	< 0.001	2.55 (1.84-3.53)
**Shock**				
No		11542	< 0.001	3.00 (2.54-3.54)
Yes		2331	< 0.001	2.35 (1.87-2.97)
**Renal disease**				
No		11379	< 0.001	2.82 (2.41-3.31)
Yes		2494	< 0.001	2.60 (1.99-3.41)

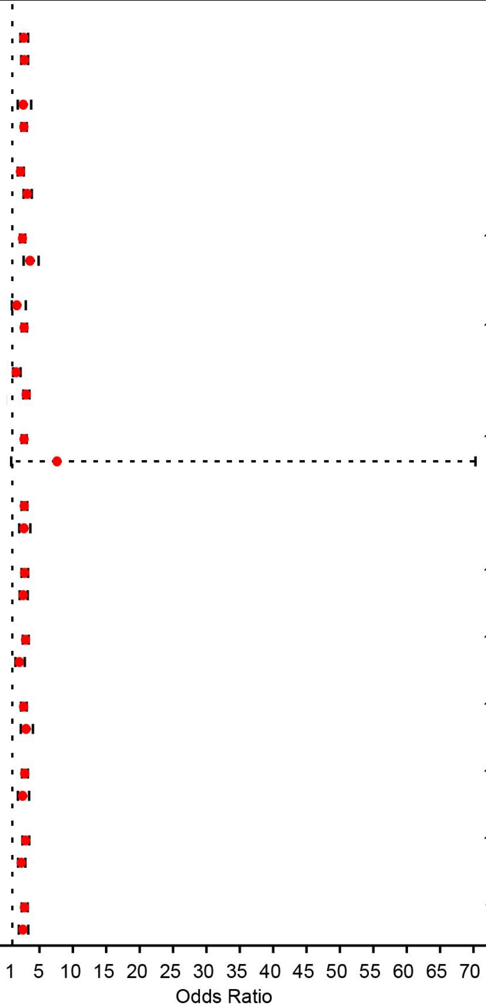

S3 Fig. Subgroup analyses of the association between SABG-3 and 28-day mortality in patients with sepsis. Subgroup analyses confirm the established model as an independent prognostic indicator. SABG-3: Sepsis assessment blood gas 3.

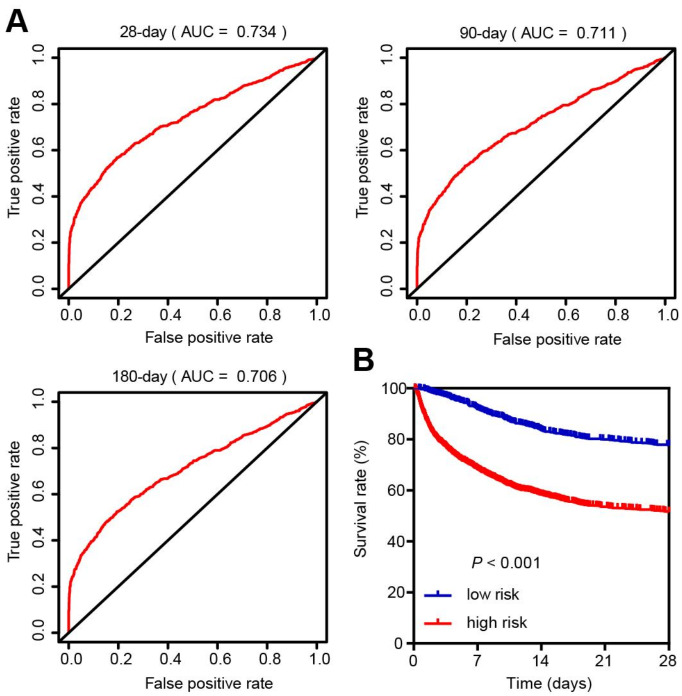

S4 Fig. Prognostic role of the blood gas parameter–based assessment model in patients with septic shock. (A) Time-dependent receiver operating characteristic curves evaluating model performance. **(B)** Kaplan–Meier survival curves for high-risk or low-risk patients with septic shock (log-rank test) as determined by the blood gas parameter–based assessment model.

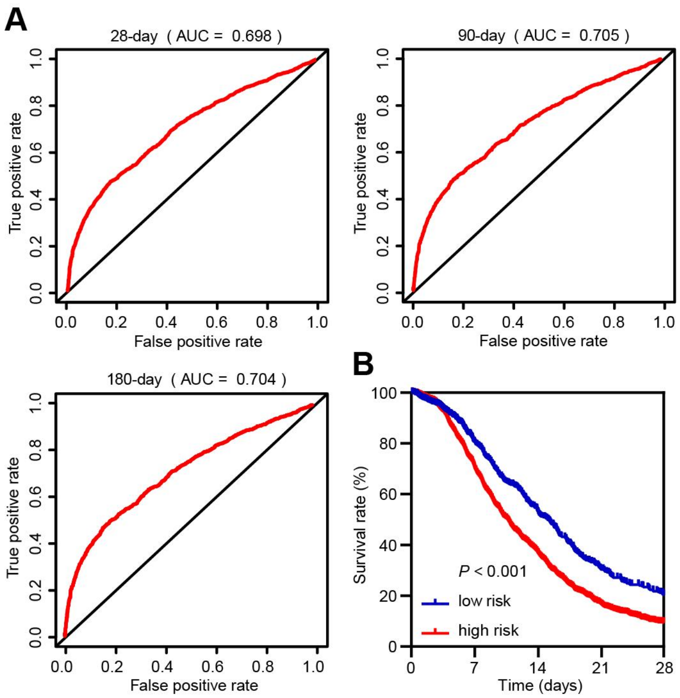

S5 Fig. Prognostic role of the blood gas parameter–based assessment model in patients with sepsis from the eICU-CRD. (A) Time-dependent receiver operating characteristic curves of the established model for evaluating its performance in patients with sepsis. **(B)** Kaplan–Meier survival curves comparing high- or low-risk patients with sepsis as determined by the blood gas parameter–based assessment model.

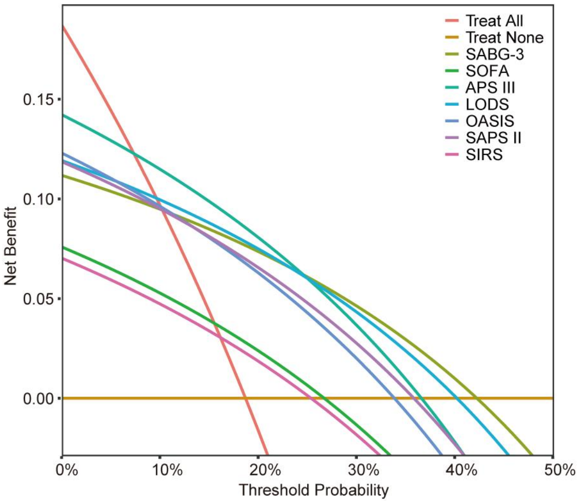

S6 Fig. Decision curve analysis of SABG-3. Decision curve analysis for comparing the net clinical benefit of SABG-3 with established sepsis severity scores. SABG-3: Sepsis assessment blood gas 3.

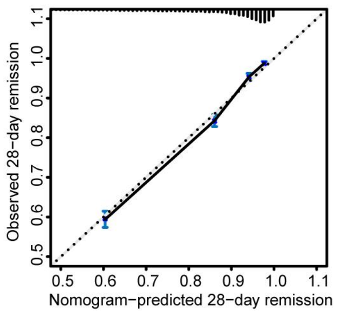

S7 Fig. Calibration plot of the SABG-3-derived nomogram. Calibration diagram was plotted to evaluate the accuracy of the constructed nomogram. SABG-3: Sepsis assessment blood gas 3.

Example code

R Codes for LASSO regression

```
library("glmnet")
library("survival")
rt=read.table(".txt",header=T,sep="\t",row.names=1,check.names=F)    rt$futime[rt$futime<=0]=1
x=as.matrix(rt[,c(3:ncol(rt))])
y=data.matrix(Surv(rt$futime,rt$fustat))
fit <- glmnet(x, y, family = "cox", maxit = 1000)
pdf("lambda.pdf")
plot(fit, xvar = "lambda", label = TRUE)
dev.off()
cvfit <- cv.glmnet(x, y, family="cox", maxit = 1000)
pdf("cvfit.pdf")
plot(cvfit)
abline(v=log(c(cvfit$lambda.min,cvfit$lambda.1se)),lty="dashed")
dev.off()
coef <- coef(fit, s = cvfit$lambda.min)
index <- which(coef != 0)
actCoef <- coef[index]
lassoGene=row.names(coef)[index]
lassoGene=c("futime","fustat",lassoGene)
lassoSigExp=rt[,lassoGene]
lassoSigExp=cbind(id=row.names(lassoSigExp),lassoSigExp)
write.table(lassoSigExp,file="lassoSigExp.txt",sep="\t",row.names=F,quote=F)
```

R Codes for propensity score IPW and doubly robust

```
library(readxl)
library(writexl)
library(drgee)
library(car)
dt <- read_xlsx('.xlsx',sheet=1,col_names = T,col_types = NULL ,na="")
dt <- as.data.frame(dt)
colnames(dt)
vartype <- read_xlsx('.xlsx',sheet=2,col_names = T,col_types = NULL ,na="")
vartype <- as.data.frame(vartype)
for (i in 1:dim(dt)[2]) {
  temp <- vartype[vartype==colnames(dt)[i],2]
  if(temp==0){dt[,i] <- dt[,i]}
  else if(temp==1){
    dt[,i] <- as.numeric(dt[,i])
  } else if(temp==2|temp==3){
```

```
        dt[,i] <- factor(dt[,i])
    }
}
f1 <- function(v){return(sum(is.na(v))*100/nrow(dt))}
re <- apply(dt, 2, f1)
re <- as.data.frame(re)
re$revar <- rownames(re)
f2 <- function(v){return(!any(is.na(v)))}
re <- as.data.frame(apply(dt, 1, f2))
sum(re[,1])
dt <- dt[re[,1],]
save(vartype,dt,file = 'data.Rdata')
```

For propensity score IPW

```
vars=colnames(dt)[-c(1:3)]
psModel=glm(risk_1~.,
            family=binomial(link="logit"),
            data=dt[,c('risk_1',vars)])
dt$ps=predict(psModel,type="response")
dt$wt1=1/dt$ps
dt$wt0=1/(1-dt$ps)
dt$w <- ifelse(dt$risk_1=="1",dt$wt1,dt$wt0)
glm.m=glm(status~risk_1,data=dt,
            family=binomial(link="logit"),
            weights=dt$w)
summary(glm.m)
re<- summary(glm.m)
re <- data.frame(round(re$coefficients,3),'OR'= round(exp(re$coefficients[,1]),3),round(exp(confint(glm.m)),3))
colnames(re)[c(4,6,7)] <-c('P','OR_95%CI_LOW','OR_95%CI_UP')
re$`OR(95%CI)` <-
paste(round(re$OR,3),'(',round(re$`OR_95%CI_LOW`,3),',',round(re$`OR_95%CI_UP`,3),')',sep='')
re$P <- round(re$P,3);re <- re[-1,]
re
write.csv(re,'Propensity score IPW.csv')
```

For doubly robust

```
vars
paste(vars,'+',sep='',collapse = '')
fit <- drgee(oformula = status ~
age+bmi+sofa+apsiii+lods+oasis+sapsii+sirs+heartrate+meanbp+resprate+temp+sex+type+race+marital+unit+rrt+v
aso+ele_surgery+ven+shock+chronic_pulmonary_disease+coronary+diabetes+hypertension+cancer+liver+congestiv
e_heart_failure+cerebrovascular_disease+renal,
,
```

```
                eformula = risk_1 ~
age+bmi+sofa+apsiii+lods+oasis+sapsii+sirs+heartrate+meanbp+resprate+temp+sex+type+race+marital+unit+rrt+v
aso+ele_surgery+ven+shock+chronic_pulmonary_disease+coronary+diabetes+hypertension+cancer+liver+congestiv
e_heart_failure+cerebrovascular_disease+renal,
                olink = "logit",
                elink = "logit",
                estimation.method = "dr",
                data = dt)
summary(fit)
re<- summary(fit)
re <- data.frame(round(re$coefficients,3),'OR'= round(exp(re$coefficients[,1]),3),round(exp(confint(fit)),3))
colnames(re)[c(4,6,7)] <-c('P','OR_95%CI_LOW','OR_95%CI_UP')
re$`OR(95%CI)` <-
paste(round(re$OR,3),'(',round(re$`OR_95%CI_LOW`,3),',',round(re$`OR_95%CI_UP`,3),')',sep='')
re$P <- round(re$P,3);
re
write.csv(re,'DoublyRobust.csv')
```

R Codes for nomograms establishment

```
library(readxl)
library(writexl)
library(rms)
dt <- read_xlsx('.xlsx',sheet=1,col_names = T,col_types = NULL ,na="")
dt <- as.data.frame(dt)[,-1]
colnames(dt)
str(dt)
vartype <- read_xlsx('.xlsx',sheet=2,col_names = T,col_types = NULL ,na="")
vartype <- as.data.frame(vartype)
for (i in 1:dim(dt)[2]) {
  temp <- vartype[vartype==colnames(dt)[i],2]
  if(temp==0){dt[,i] <- dt[,i]}
  else if(temp==1){
    dt[,i] <- as.numeric(dt[,i])
  } else if(temp==2|temp==3){
    dt[,i] <- factor(dt[,i])
  }
}
f1 <- function(v){return(sum(is.na(v))*100/nrow(dt))}
re <- apply(dt, 2, f1)
re <- as.data.frame(re)
re$revar <- rownames(re)
```

```
f2 <- function(v){return(!any(is.na(v)))}
re <- as.data.frame(apply(dt, 1, f2))
sum(re[,1])
dt <- dt[re[,1],]
save(vartype,dt,file = 'data.Rdata')
get(load(file = 'data.Rdata'))
str(dt)
rt <- dt
rt$futime=rt$futime/1
dd <- datadist(rt)
options(datadist="dd")
paste(colnames(rt),collapse = '+')
f <- cph(Surv(futime, fustat) ~
Age+BMI+Admission_type+Service_unit+RRT_use+Norepinephrine_use+Elective_surgery+Mechanical_ventilatio
n_use+Cancer+Liver_disease+RiskScore, x=T, y=T, surv=T, data=rt)
surv <- Survival(f)
nom <- nomogram(f, fun=list(function(x) surv(1, x),
                            function(x) surv(14, x),
                            function(x) surv(28, x)),
                lp=F,
                funlabel=c("1-day survival", "14-day survival", "28-day survival"),
                maxscale=100,
                fun.at=c(0.99, 0.9, 0.8, 0.7, 0.6, 0.4,0.2,0.05))
pdf(file="nomogram.pdf",height=11,width=11)
plot(nom,
     cex.axis=0.6,
     cex.var=0.7,
     ia.space  =0.5)
dev.off()
time = 1
cox1 <- cph(Surv(futime, fustat) ~
Age+BMI+Admission_type+Service_unit+RRT_use+Norepinephrine_use+Elective_surgery+Mechanical_ventilatio
n_use+Cancer+Liver_disease+RiskScore, x=T, y=T, surv=T, data=rt, time.inc=time)
cal <- calibrate(cox1, cmethod="KM", method="boot", u=time, m= round(nrow(rt)/5,0), B=1000)
par(mar = c(10,5,3,2),cex = 1.0)
road <- file.path('1.day.cali.pdf')
pdf(file=road,height=11,width=11,pointsize = 14)
plot(cal,lwd=3,lty=2,subtitles=F,
     errbar.col=c(rgb(0,118,192,maxColorValue = 255)),
     xlim = c(0.94,1.01),
     ylim = c(0.94,1.01),
```

```
        conf.int=T,
        xlab = "Nomogram-predicted 1-day remission (probability)",
        ylab="Observed 1-day remission (probability)",
        col="black",
        cex.axis=1,
        cex.subtitles=.65)
lines(cal[,c('mean.predicted','KM')],
          type = 'l',lwd = 2,
          col ="black" ,pch = 16)
box(lwd = 1)
abline(0,1,lty = 3,lwd = 1,col = "black")
dev.off()
time = 14
cox1 <- cph(Surv(futime, fustat) ~
Age+BMI+Admission_type+Service_unit+RRT_use+Norepinephrine_use+Elective_surgery+Mechanical_ventilatio
n_use+Cancer+Liver_disease+RiskScore, x=T, y=T, surv=T, data=rt, time.inc=time)
cal <- calibrate(cox1, cmethod="KM", method="boot", u=time, m= round(nrow(rt)/5,0), B=1000)
par(mar = c(10,5,3,2),cex = 1.0)
road <- file.path('14.day.cali.pdf')
pdf(file=road,height=11,width=11,pointsize = 14)
plot(cal,lwd=3,lty=2,subtitles=F,
        errbar.col=c(rgb(0,118,192,maxColorValue = 255)),
        xlim = c(0.5,1.1),
        ylim = c(0.5,1.1),
        conf.int=T,
        xlab = "Nomogram-predicted 14-day remission (probability)",
        ylab="Observed 14-day remission (probability)",
        col="black",
        cex.subtitles=.65)
lines(cal[,c('mean.predicted','KM')],
          type = 'l',lwd = 2,
          col ="black" ,pch = 16)
box(lwd = 1)
abline(0,1,lty = 3,lwd = 1,col = "black")
dev.off()
time = 28
cox1 <- cph(Surv(futime, fustat) ~
Age+BMI+Admission_type+Service_unit+RRT_use+Norepinephrine_use+Elective_surgery+Mechanical_ventilatio
n_use+Cancer+Liver_disease+RiskScore, x=T, y=T, surv=T, data=rt, time.inc=time)
cal <- calibrate(cox1, cmethod="KM", method="boot", u=time, m= round(nrow(rt)/5,0), B=1000)
par(mar = c(10,5,3,2),cex = 1.0)
```

```
road <- file.path('28.day.cali.pdf')
pdf(file=road,height=11,width=11,pointsize = 14)
plot(cal,lwd=3,lty=2,subtitles=F,
     errbar.col=c(rgb(0,118,192,maxColorValue = 255)),
     xlim = c(0.5,1.1),
     ylim = c(0.5,1.1),
     conf.int=T,
     xlab = "Nomogram-predicted 28-day remission (probability)",
     ylab="Observed 28-day remission (probability)",
     col="black",
     cex.subtitles=.65)
lines(cal[,c('mean.predicted','KM')],
      type = 'l',lwd = 2,
      col ="black" ,pch = 16)
box(lwd = 1)
abline(0,1,lty = 3,lwd = 1,col = "black")
dev.off()
```

Brief descriptions of each method

Methods	Descriptions
LASSO regression	LASSO is a variable selection method. Compared to traditional regression approaches, LASSO regression can handle a larger set of potential predictors and select the variables most associated with the disease.
Propensity score-based matching	A method used to balance important confounding factors between different groups.
Propensity score inverse probability weighting	A method used to correct both confounding and forms of selection bias, such as informative censoring.
Doubly robust	A method that combines a multivariable regression model with a propensity score model to estimate the association and causal effect of an exposure on an outcome.
Nomogram	A graphical prediction model that integrates multiple quantitative and qualitative variables to predict the occurrence of a specific event. It can use an intuitive mapping to assess the risk of an individual patient.
