# Supplementary material for: A blood gas parameter–based assessment model for predicting poor prognosis in sepsis: A retrospective analysis of the MIMIC-IV and eICU-CRD
Source: PLoS One. 2026 Jul 9;21(7):e0346532. doi: 10.1371/journal.pone.0346532 (PMC13349094; doi:10.1371/journal.pone.0346532)
Supplement: S8 Table — (PDF) [file pone.0346532.s008.pdf]

S8 Table. Impact of the SABG-3-derived risk score and other important variables on 28-day mortality in patients with sepsis from the eICU-CRD.

Variables	Univariable models		Full multivariable model	
	**Odds ratio (95% CI)**	***P* value**	**Odds ratio (95% CI)**	***P* value**
SABG-3-derived risk score	21.651 (15.815-29.641)	<.001	301.886 (2.166-42082.601)	0.023
Age	0.984 (0.980-0.989)	<.001	1.018 (0.935-1.108)	0.682
Gender (Male)	0.910 (0.796-1.040)	0.167	0.194 (0.027-1.400)	0.104
BMI	1.000 (1.000-1.000)	0.782	0.988 (0.877-1.114)	0.846
Admission type	1.343 (1.175-1.535)	<.001	2.351 (0.184-30.107)	0.511
Race				
White	Reference		Reference	
Asian	0.969 (0.719-1.306)	0.835	0.194 (0.001-68.570)	0.584
Black	0.873 (0.512-1.490)	0.618	0.085 (0.000-76.765)	0.478
Hispanic	0.827 (0.577-1.186)	0.301	0.013 (0.000-11.419)	0.210
Other	1.142 (0.680-1.916)	0.616	/	1.000
Service unit	0.903 (0.739-1.103)	0.319	0.009 (0.000-14.899)	0.214
Severity of illness				
SOFA score	0.826 (0.811-0.842)	<.001	0.841 (0.534-1.324)	0.454
SAPS II score	0.948 (0.943-0.952)	<.001	0.992 (0.854-1.152)	0.917
OASIS score	0.945 (0.938-0.951)	<.001	0.992 (0.854-1.152)	0.917
APS III score	0.983 (0.981-0.985)	<.001	1.018 (0.983-1.054)	0.315
LODS score	0.812 (0.796-0.828)	<.001	1.208 (0.774-1.886)	0.406
SIRS score	0.622 (0.572-0.678)	<.001	0.661 (0.145-3.016)	0.593
Interventions				
RRT use	0.384 (0.312-0.471)	<.001	0.462 (0.018-11.643)	0.639
Mechanical ventilation use	0.386 (0.336-0.444)	<.001	0.046 (0.003-0.786)	0.034
Vasopressor use	0.499 (0.436-0.571)	<.001	17.539 (1.599-192.401)	0.019
Elective surgery	0.973 (0.694-1.364)	0.872	0.128 (0.007-2.314)	0.164
Comorbidities				
Hypertension	1.266 (0.992-1.616)	0.058	0.354 (0.018-6.975)	0.495
Diabetes	1.223 (0.945-1.584)	0.126	1.326 (0.020-89.999)	0.896
CPD	0.972 (0.750-1.259)	0.831	0.233 (0.007-8.204)	0.422
Coronary	0.938 (0.588-1.497)	0.787	/	0.998
CHF	0.844 (0.554-1.285)	0.429	/	/
Cancer	0.656 (0.494-0.872)	0.004	1.369 (0.28-67.000)	0.874
Liver disease	0.445 (0.334-0.594)	<.001	23.199 (0.462-1166.077)	0.116
Renal disease	0.623 (0.496-0.782)	<.001	/	0.998
Cerebrovascular disease	0.475 (0.295-0.766)	0.002	/	1.000
Shock	0.579 (0.495-0.677)	<.001	0.617 (0.075-5.078)	0.653
Vital signs				
MAP (mmHg)	1.011 (0.999-1.023)	0.067	1.057 (0.999-1.118)	0.055
Heart rate (bpm)	0.999 (0.995-1.002)	0.480	0.995 (0.937-1.057)	0.880
Temperature (℃)	1.123 (1.026-1.228)	0.011	1.186 (0.469-3.002)	0.718
Respiratory rate (bpm)	0.986 (0.975-0.996)	0.009	1.050 (0.908-1.215)	0.512

BMI: Body mass index

SOFA: Sequential organ failure assessment

SAPS II: Simplified acute physiology score II

OASIS: Oxford acute severity of illness score

APS III: Acute physiology score III

LODS: Logistic organ dysfunction system

SIRS: Systemic inflammatory response syndrome

RRT: Renal replacement therapy

CPD: Chronic pulmonary disease
CHF: Congestive heart failure
MAP: Mean arterial pressure
