# Supplementary material for: A blood gas parameter–based assessment model for predicting poor prognosis in sepsis: A retrospective analysis of the MIMIC-IV and eICU-CRD
Source: PLoS One. 2026 Jul 9;21(7):e0346532. doi: 10.1371/journal.pone.0346532 (PMC13349094; doi:10.1371/journal.pone.0346532)
Supplement: S9 Table — (PDF) [file pone.0346532.s009.pdf]

S9 Table. Details of the doubly robust model evaluating the effect of variables on 28-day mortality.

Variables	Odds ratio (95% CI)	*P* value
Age	1.002 (1.002-1.003)	<.001
Body mass index	0.998 (0.997-0.998)	<.001
Admission type	1.583 (1.339-1.872)	<.001
Service unit	1.238 (1.087-1.411)	0.001
Renal replacement therapy	1.254 (1.058-1.487)	0.009
Norepinephrine	1.737 (1.496-2.017)	<.001
Elective surgery	0.319 (0.171-0.592)	<.001
Mechanical ventilation	1.628 (1.221-2.171)	0.001
Cancer	1.544 (1.298-1.837)	<.001
Liver disease	1.340 (1.145-1.569)	<.001
