# Supplementary material for: A blood gas parameter–based assessment model for predicting poor prognosis in sepsis: A retrospective analysis of the MIMIC-IV and eICU-CRD
Source: PLoS One. 2026 Jul 9;21(7):e0346532. doi: 10.1371/journal.pone.0346532 (PMC13349094; doi:10.1371/journal.pone.0346532)
Supplement: S1 Fig — Time-dependent receiver operating characteristic curves for arterial (upper) and venous (below) serum lactate levels. (PDF) [file pone.0346532.s010.pdf]

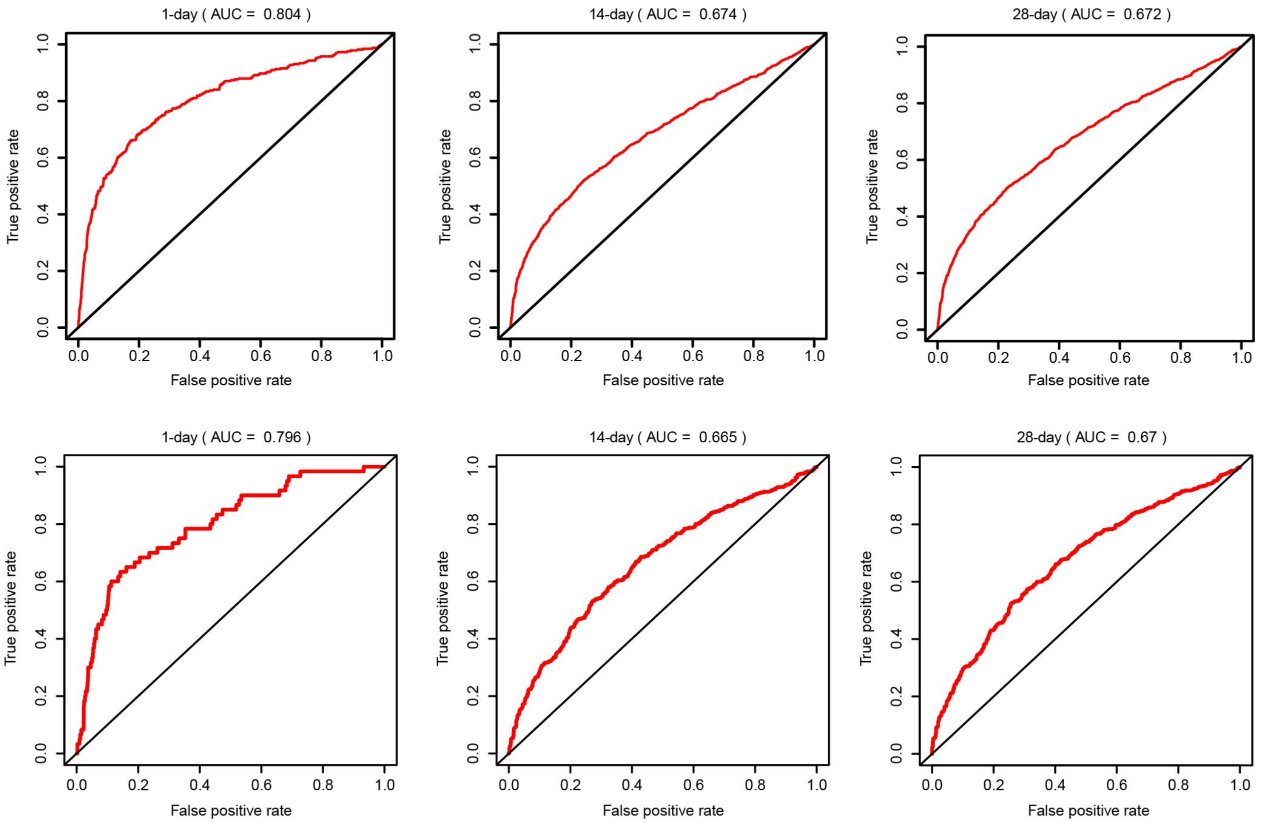

S1 Fig. Prognostic performance of serum lactate in patients with sepsis. Time-dependent receiver operating characteristic curves for arterial (upper) and venous (below) serum lactate levels.
