# Supplementary material for: A blood gas parameter–based assessment model for predicting poor prognosis in sepsis: A retrospective analysis of the MIMIC-IV and eICU-CRD
Source: PLoS One. 2026 Jul 9;21(7):e0346532. doi: 10.1371/journal.pone.0346532 (PMC13349094; doi:10.1371/journal.pone.0346532)
Supplement: S2 Fig — Propensity score distribution or absolute standardized mean differences confirm negligible variance between the control (low-risk) and treatment (high-risk) groups after propensity score matching. (PDF) [file pone.0346532.s011.pdf]

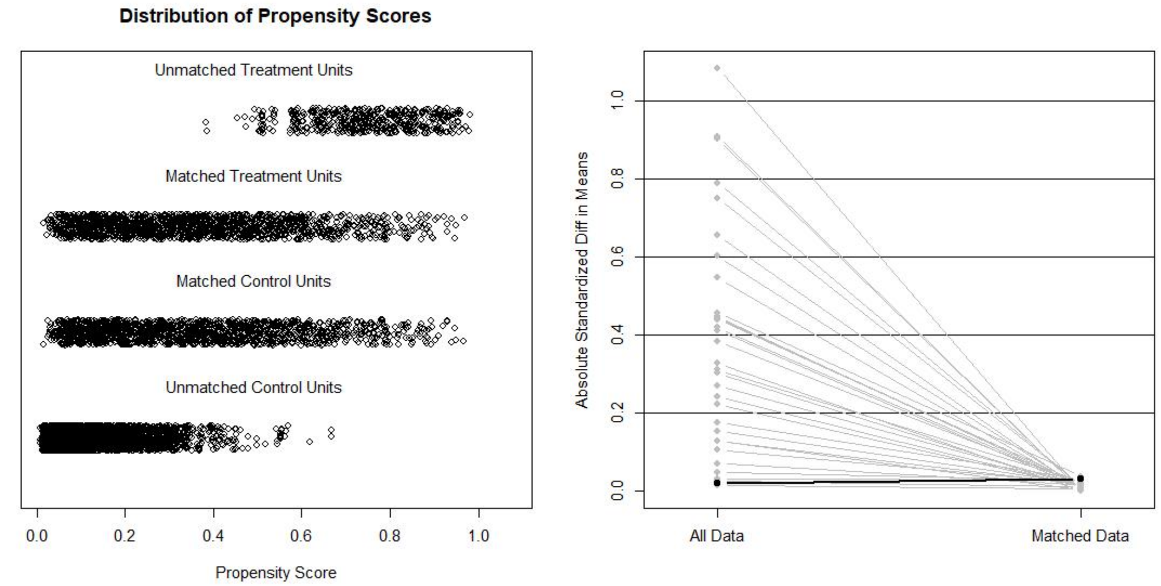

S2 Fig. Balanced distribution of baseline characteristics between high-risk and low-risk groups after propensity score matching. Propensity score distribution or absolute standardized mean differences confirm negligible variance between the control (low-risk) and treatment (high-risk) groups after propensity score matching.
