# Supplementary material for: A blood gas parameter–based assessment model for predicting poor prognosis in sepsis: A retrospective analysis of the MIMIC-IV and eICU-CRD
Source: PLoS One. 2026 Jul 9;21(7):e0346532. doi: 10.1371/journal.pone.0346532 (PMC13349094; doi:10.1371/journal.pone.0346532)
Supplement: S3 Fig — Subgroup analyses confirm the established model as an independent prognostic indicator. SABG-3: Sepsis assessment blood gas 3. (PDF) [file pone.0346532.s012.pdf]

Subgroup	High risk determined by SABG-3	No of patients	*P* value	Odds Ratio (95% CI)
**Gender**				
Female		5402	< 0.001	2.74 (2.23-3.38)
Male		8471	< 0.001	2.83 (2.36-3.38)
**Admission type**				
Elective		4008	< 0.001	2.65 (1.83-3.84)
Emergency/Urgent		9865	< 0.001	2.74 (2.37-3.17)
**Service unit**				
Medical intensive care unit		4257	< 0.001	2.23 (1.82-2.71)
Others		9616	< 0.001	3.25 (2.69-3.92)
**Renal replacement therapy**				
No		12388	< 0.001	2.53 (2.17-2.95)
Yes		1485	< 0.001	3.66 (2.71-4.96)
**Mechanical ventilation**				
No		2556	0.081	1.69 (0.94-3.04)
Yes		11317	< 0.001	2.78 (2.42-3.19)
**Vasopressor**				
No		4183	0.008	1.59 (1.13-2.25)
Yes		9690	< 0.001	3.08 (2.66-3.58)
**Elective surgery**				
No		12992	< 0.001	2.77 (2.42-3.17)
Yes		881	0.071	7.70 (0.84-70.39)
**Hypertension**				
No		9357	< 0.001	2.80 (2.40-3.26)
Yes		4516	< 0.001	2.75 (2.04-3.71)
**Congestive heart failure**				
No		10017	< 0.001	2.85 (2.40-3.38)
Yes		3856	< 0.001	2.65 (2.11-3.31)
**Cancer**				
No		12190	< 0.001	2.98 (2.57-3.47)
Yes		1683	< 0.001	2.07 (1.49-2.88)
**Liver disease**				
No		11835	< 0.001	2.70 (2.31-3.15)
Yes		2038	< 0.001	3.07 (2.30-4.09)
**Cerebrovascular disease**				
No		11876	< 0.001	2.87 (2.47-3.33)
Yes		1997	< 0.001	2.55 (1.84-3.53)
**Shock**				
No		11542	< 0.001	3.00 (2.54-3.54)
Yes		2331	< 0.001	2.35 (1.87-2.97)
**Renal disease**				
No		11379	< 0.001	2.82 (2.41-3.31)
Yes		2494	< 0.001	2.60 (1.99-3.41)

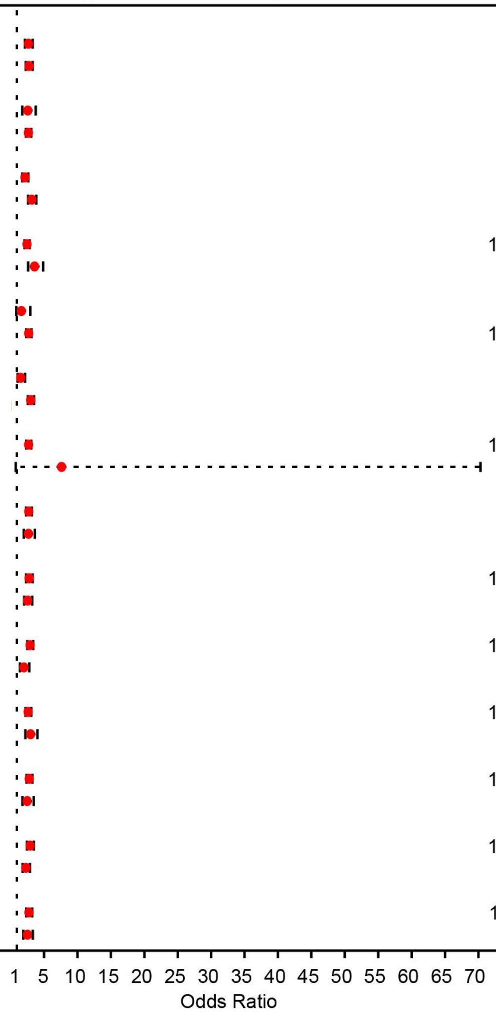

S3 Fig. Subgroup analyses of the association between SABG-3 and 28-day mortality in patients with sepsis. Subgroup analyses confirm the established model as an independent prognostic indicator. SABG-3: Sepsis assessment blood gas 3.
