# Supplementary material for: A blood gas parameter–based assessment model for predicting poor prognosis in sepsis: A retrospective analysis of the MIMIC-IV and eICU-CRD
Source: PLoS One. 2026 Jul 9;21(7):e0346532. doi: 10.1371/journal.pone.0346532 (PMC13349094; doi:10.1371/journal.pone.0346532)
Supplement: S4 Fig — (A) Time-dependent receiver operating characteristic curves evaluating model performance. (B) Kaplan–Meier survival curves for high-risk or low-risk patients with septic shock (log-rank test) as determined by the blood gas parameter–based assessment model. (PDF) [file pone.0346532.s013.pdf]

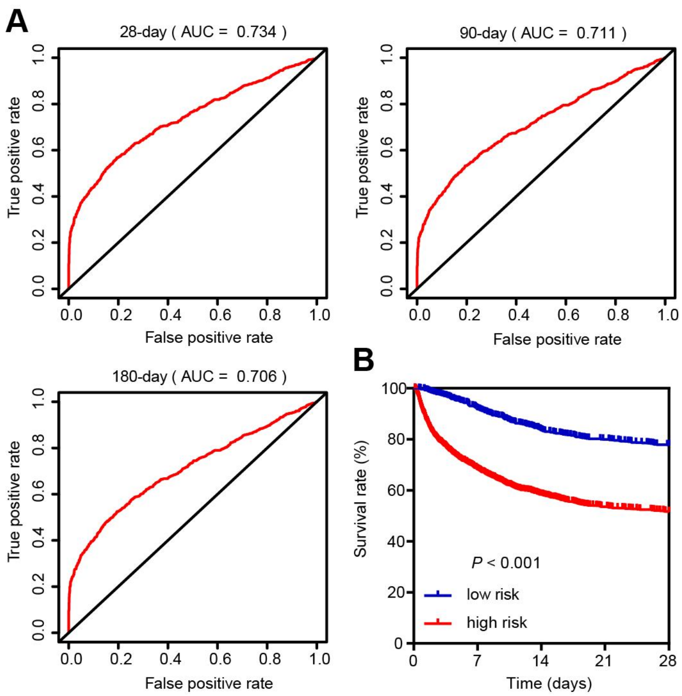

S4 Fig. Prognostic role of the blood gas parameter–based assessment model in patients with septic shock. (A) Time-dependent receiver operating characteristic curves evaluating model performance. **(B)** Kaplan–Meier survival curves for high-risk or low-risk patients with septic shock (log-rank test) as determined by the blood gas parameter–based assessment model.
