# Supplementary material for: A blood gas parameter–based assessment model for predicting poor prognosis in sepsis: A retrospective analysis of the MIMIC-IV and eICU-CRD
Source: PLoS One. 2026 Jul 9;21(7):e0346532. doi: 10.1371/journal.pone.0346532 (PMC13349094; doi:10.1371/journal.pone.0346532)
Supplement: S6 Fig — Decision curve analysis for comparing the net clinical benefit of SABG-3 with established sepsis severity scores. SABG-3: Sepsis assessment blood gas 3. (PDF) [file pone.0346532.s015.pdf]

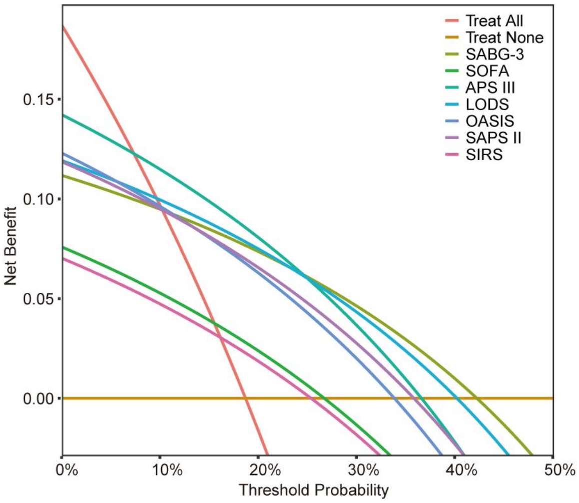

S6 Fig. Decision curve analysis of SABG-3. Decision curve analysis for comparing the net clinical benefit of SABG-3 with established sepsis severity scores. SABG-3: Sepsis assessment blood gas 3.
