# Supplementary material for: A blood gas parameter–based assessment model for predicting poor prognosis in sepsis: A retrospective analysis of the MIMIC-IV and eICU-CRD
Source: PLoS One. 2026 Jul 9;21(7):e0346532. doi: 10.1371/journal.pone.0346532 (PMC13349094; doi:10.1371/journal.pone.0346532)
Supplement: S7 Fig — Calibration diagram was plotted to evaluate the accuracy of the constructed nomogram. SABG-3: Sepsis assessment blood gas 3. (PDF) [file pone.0346532.s016.pdf]

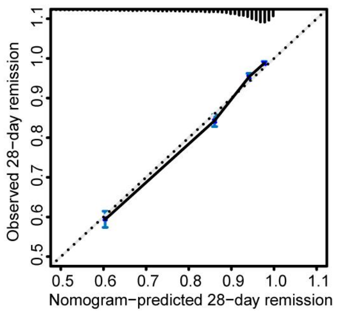

S7 Fig. Calibration plot of the SABG-3-derived nomogram. Calibration diagram was plotted to evaluate the accuracy of the constructed nomogram. SABG-3: Sepsis assessment blood gas 3.
